# More than *mcr*: canonical plasmid- and transposon-encoded mobilized colistin resistance genes represent a subset of phosphoethanolamine transferases

**DOI:** 10.3389/fcimb.2023.1060519

**Published:** 2023-06-08

**Authors:** Ahmed Gaballa, Martin Wiedmann, Laura M. Carroll

**Affiliations:** ^1^ Department of Food Science, Cornell University, Ithaca, NY, United States; ^2^ Department of Clinical Microbiology, SciLifeLab, Umeå University, Umeå, Sweden; ^3^ Laboratory for Molecular Infection Medicine Sweden (MIMS), Umeå University, Umeå, Sweden; ^4^ Umeå Centre for Microbial Research, Umeå University, Umeå, Sweden; ^5^ Integrated Science Lab, Umeå University, Umeå, Sweden

**Keywords:** MCR, colistin, phosphoethanolamine transferase, antimicrobial resistance, horizontal gene transfer, mobile genetic element, plasmid

## Abstract

Mobilized colistin resistance genes (*mcr*) may confer resistance to the last-resort antimicrobial colistin and can often be transmitted horizontally. *mcr* encode phosphoethanolamine transferases (PET), which are closely related to chromosomally encoded, intrinsic lipid modification PET (i-PET; e.g., EptA, EptB, CptA). To gain insight into the evolution of *mcr* within the context of i-PET, we identified 69,814 MCR-like proteins present across 256 bacterial genera (obtained by querying known MCR family representatives against the National Center for Biotechnology Information [NCBI] non-redundant protein database via protein BLAST). We subsequently identified 125 putative novel *mcr*-like genes, which were located on the same contig as (i) ≥1 plasmid replicon and (ii) ≥1 additional antimicrobial resistance gene (obtained by querying the PlasmidFinder database and NCBI’s National Database of Antibiotic Resistant Organisms, respectively, via nucleotide BLAST). At 80% amino acid identity, these putative novel MCR-like proteins formed 13 clusters, five of which represented putative novel MCR families. Sequence similarity and a maximum likelihood phylogeny of *mcr*, putative novel *mcr*-like, and *ipet* genes indicated that sequence similarity was insufficient to discriminate *mcr* from *ipet* genes. A mixed-effect model of evolution (MEME) indicated that site- and branch-specific positive selection played a role in the evolution of alleles within the *mcr-2* and *mcr-9* families. MEME suggested that positive selection played a role in the diversification of several residues in structurally important regions, including (i) a bridging region that connects the membrane-bound and catalytic periplasmic domains, and (ii) a periplasmic loop juxtaposing the substrate entry tunnel. Moreover, *eptA* and *mcr* were localized within different genomic contexts. Canonical *eptA* genes were typically chromosomally encoded in an operon with a two-component regulatory system or adjacent to a TetR-type regulator. Conversely, *mcr* were represented by single-gene operons or adjacent to *pap2* and *dgkA*, which encode a PAP2 family lipid A phosphatase and diacylglycerol kinase, respectively. Our data suggest that *eptA* can give rise to “colistin resistance genes” through various mechanisms, including mobilization, selection, and diversification of genomic context and regulatory pathways. These mechanisms likely altered gene expression levels and enzyme activity, allowing *bona fide eptA* to evolve to function in colistin resistance.

## 1 Introduction

Colistin, a polycationic peptide, serves as a last-resort antimicrobial, which can be used to treat infections caused by multidrug-resistant (MDR), extensively drug-resistant (XDR), and pan-drug-resistant (PDR) Gram-negative bacteria (El-Sayed Ahmed et al., 2020; Binsker et al., 2022). Consequently, colistin has been designated by the World Health Organization (WHO) as a highest priority critically important antimicrobial for human medicine (World Health Organization, 2019). Due to the critical importance of colistin as an antibiotic of last resort, colistin resistance among Gram-negative pathogens represents an increasingly dire global public health threat (El-Sayed Ahmed et al., 2020; World Health Organization, 2021; Binsker et al., 2022).

**Figure 1:**
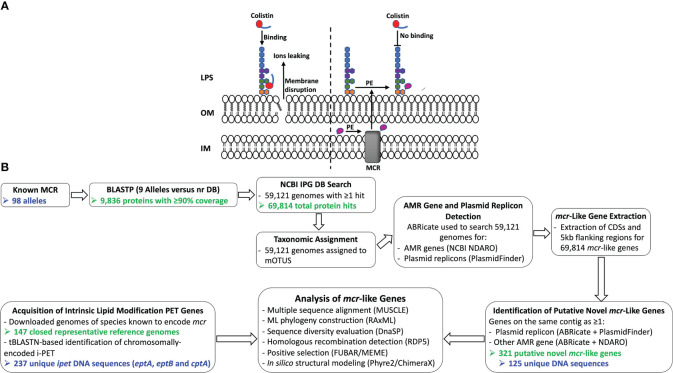
**(A)** Schematic diagram of colistin’s mode of action and resistance mechanism. Colistin, a cationic antimicrobial peptide, binds to the negatively charged lipid A, displacing membrane-bound cations and disrupting membrane integrity by inserting the hydrophobic tail into the membrane’s lipid (panel A, left). Lipid A modification neutralizes the negative membrane charge, thus reducing colistin binding and cell susceptibility to colistin (panel A, right). IM, inner membrane; LPS, lipopolysaccharide; MCR, mobilized colistin resistance protein; OM, outer membrane; PE, phosphoethanolamine. **(B)** Schematic outline of the main methodologies used to acquire and characterize MCR and MCR-like proteins from publicly available whole-genome sequencing (WGS) data (see the Materials and Methods section for details). The number of MCR, putative novel MCR-like, and/or intrinsic lipid modification phosphoethanolamine transferase (i-PET) proteins produced at relevant steps are shown in green text; the number of such genes/proteins used in final analyses are shown in blue text. AMR, antimicrobial resistance; BLASTP, protein basic local alignment search tool; CDSs, coding sequences; DB, database; IPG, Identical Protein Group; LPS, lipopolysaccharide; MCR, mobilized colistin resistance amino acid sequences; ML, maximum likelihood; mOTUs, marker gene-based operational taxonomic units; NCBI, National Center for Biotechnology Information; NDARO, National Database of Antibiotic Resistant Organisms; nr, non-redundant protein sequence; (i) -PET, (intrinsic) phosphoethanolamine transferases; tBLASTN, translated nucleotide basic local alignment search tool.

The binding of colistin to bacterial cells is initiated by an electrostatic attraction between the colistin cationic head group and the anionic phosphate group on the lipid A of the bacterial lipopolysaccharide (LPS), which displaces Ca^2+^ and Mg^2+^ ions (Olaitan et al., 2014; El-Sayed Ahmed et al., 2020). Subsequently, colistin’s hydrophobic tail integration in the lipid bilayers leads to cell membrane disruption and cell death by targeting cytoplasmic membrane LPS (Trimble et al., 2016; Sabnis et al., 2021) (**Figure 1A**). Multiple colistin resistance mechanisms have been reported, including (i) modification of lipid A (Guo et al., 1997; Gunn et al., 1998); (ii) expression of a multi-drug efflux system in *Pseudomonas aeruginosa* (Abuoun et al., 2017); (iii) the complete loss of LPS in *Acinetobacter baumannii* (Moffatt et al., 2010); (iv) overproduction of capsular polysaccharide in *Klebsiella pneumoniae* (Campos et al., 2004); and (v) enzymatic degradation of colistin via colistin-degrading proteases in *Stenotrophomonas maltophilia* (Ito-Kagawa and Koyama, 1980; Lee et al., 2022). The main mechanism of bacterial colistin resistance is the modification of lipid A via the addition of a cationic group, such as phosphoethanolamine (pEtN), 4-amino-4-deoxy-L-arabinose, and glycine to lipid A (Trimble et al., 2016), which reduces the overall membrane negative charge and subsequently decreases colistin binding affinity to the cell (Olaitan et al., 2014; El-Sayed Ahmed et al., 2020). While it has been shown that lipid A is essential for growth in most bacterial species, lipid A modifications are dispensable for cell survival under laboratory growth conditions (Trent, 2004; Raetz et al., 2007; Parsons and Rock, 2013). However, lipid A modification plays a significant role in bacterial adaptation to different stress conditions, including mild acid stress, change in oxygen level, increase in growth temperature, pH change, osmotic stress, and the presence of cationic antimicrobial compounds (Trent, 2004; Raetz et al., 2007; Gunn, 2008; Anandan and Vrielink, 2020a; Troudi et al., 2021).

Some Gram-negative bacteria (e.g., *Neisseria, Serratia*, *Brucella, Burkholderia* spp.) showcase intrinsic resistance to colistin; others, including pathogenic Enterobacteriaceae, can acquire resistance via numerous mechanisms, including chromosomal mutations (e.g., those that modify the bacterial cell surface) and/or mobilized colistin resistance (*mcr*) genes (El-Sayed Ahmed et al., 2020; World Health Organization, 2021; Binsker et al., 2022). *mcr* genes represent a particularly notable and concerning mechanism of colistin resistance, as they are predominantly plasmid-borne and/or associated with transposon insertional elements and can facilitate the acquisition and rapid dissemination of colistin resistance (Kieffer et al., 2017; Trebosc et al., 2019; El-Sayed Ahmed et al., 2020). *mcr* encodes an inner membrane-anchored protein that belongs to a complex group of phosphoethanolamine transferase (PET) enzymes (Gao et al., 2016), which add phosphoethanolamine (pEtN) to the 4’ phosphate group of lipid A (Cox et al., 2003; Reynolds et al., 2005; Tamayo et al., 2005b; Raetz et al., 2007; Parsons and Rock, 2013; Anandan et al., 2017). Gram-negative bacterial species possess one or more chromosomal PET genes, which function in intrinsic lipid modification, including EptA, EptB, and CptA (Anandan and Vrielink, 2020a). For example, *Escherichia coli* str. K-12 substr. MG1655 harbors homologs of EptA, EptB, and CptA (Reynolds et al., 2005; Zhao et al., 2019; Anandan and Vrielink, 2020b). We will collectively refer to *eptA*, *eptB*, and *cptA* genes as *ipet* (intrinsic lipid modification PET) genes and their products as i-PET enzymes or proteins. Lipid A modifying i-PET enzymes phylogenetically cluster into three distinct groups that correlate with the enzyme specificity to the pEtN-acceptor substrate site on the LPS. More specifically, (i) EptA adds pEtN to the N-acetylglucosamine moiety of lipid A, (ii) EptB adds pEtN to the 3-deoxy-d-manno-octulosonic acid (KDO) of the LPS core region, and (iii) CptA/EptC adds pEtN to the second heptose of the LPS core region (Harper et al., 2017; Anandan and Vrielink, 2020a).

Interestingly, MCR proteins share a high degree of sequence and structural similarity with i-PET enzymes present in many Gram-negative bacteria (Bakovic et al., 2007; Anandan and Vrielink, 2020a; Samantha and Vrielink, 2020), including pathogenic members of Enterobacteriaceae (El-Sayed Ahmed et al., 2020; Gogry et al., 2021). PET protein structure includes two discretely folded domains connected by a bridging helix and extended loop, an N-terminal transmembrane domain, and a C-terminal periplasmic soluble catalytic domain (Anandan et al., 2017; Anandan and Vrielink, 2020a). Moreover, PET proteins are metalloenzymes that share a conserved zinc-binding site tetrahedrally coordinated by the side chains of conserved Glu^246^, Thr^285^, His^466^, and Asp^465^ residues (numbers corresponding to the MCR-1 sequence), with the Thr^285^ residue acting as the catalytic nucleophile for the pEtN transfer (Stojanoski et al., 2016; Anandan et al., 2017). While multiple conserved or partially conserved amino acid (AA) residues were suggested to be involved in pEtN binding (Sun et al., 2017; Dortet et al., 2018; Stogios et al., 2018; Carroll et al., 2019; Garcia-Menino et al., 2020), the lipid-binding pocket remains poorly defined (Carroll et al., 2019).

As of April 2022, ten major groups of MCR proteins have been defined based on their AA identities/similarities (termed MCR-1 to -10, which are encoded by *mcr* genes *mcr-1* to *-10*, respectively) (Liu et al., 2016; Xavier et al., 2016; Abuoun et al., 2017; Borowiak et al., 2017; Carattoli et al., 2017; Yin et al., 2017; Wang et al., 2018b; Yang et al., 2018; Carroll et al., 2019; Wang et al., 2020). Here, we will refer to these major groups as “*mcr* families” or “MCR families” (for nucleotide sequences/genes and amino acid sequences/proteins, respectively), as has been proposed previously (Partridge et al., 2018). Within MCR families, AA sequence identities and similarities can vary. Here, we will use the term “*mcr* allele” or “MCR allele” to denote specific variants of genes and proteins within a given MCR family, respectively (e.g., alleles within the *mcr-3* family are denoted as *mcr-3.1, mcr-3.2, mcr-3.3*, and so on, and their products as MCR-3.1, MCR-3.2, MCR-3.3, and so on, respectively) (Partridge et al., 2018). In total, >95 MCR alleles have been described as of April 2022.

While MCR proteins share a conserved overall structure (Stojanoski et al., 2016; Sun et al., 2017; Xu et al., 2018a; Carroll et al., 2019; Son et al., 2019), the genes that encode them vary in terms of sequence similarity (Li et al., 2018; Carroll et al., 2019; Ramaloko and Osei Sekyere, 2022). Moreover, it has been suggested that *mcr* alleles can vary in terms of the colistin resistance levels they confer (Nang et al., 2019; El-Sayed Ahmed et al., 2020). However, the molecular bases of *mcr* genetic and phenotypic heterogeneities are poorly understood. Furthermore, it has been suggested that *mcr* evolved from an *eptA* chromosomal gene copy via mobilization and that *Moraxella* spp. are a potential source for MCR-like colistin resistance determinants (Kieffer et al., 2017). Indeed, several lines of evidence support the notion that *mcr* evolved from *eptA* through mobilization, including: (i) MCR and EptA share a conserved overall protein structure and acceptor substrate specificity, (ii) IS*Apl1*-dependent mobilization of *mcr* has been demonstrated (Abuoun et al., 2017; Kieffer et al., 2017), and (iii) insertion of IS*Apl1* upstream of *eptA* increased *eptA* expression and led to an increased level of colistin resistance in XDR *Acinetobacter baumannii* isolates (Trebosc et al., 2019). However, the exact ancestor of *mcr* and the molecular mechanism of evolution of intrinsic lipid modification *eptA* to mobilized colistin-resistant determinant remain unknown.

Here, we aim to provide further insight into the evolutionary relationships between *mcr* families, all within the context of *ipet*. We show that some *ipet* genes (e.g., *eptA*) may differ from canonical *mcr* in terms of their genomic context, and we identify differentially conserved residues, which may play a role in the levels of colistin resistance conferred by different *mcr* alleles. Finally, using a large number (>69,000) of MCR and MCR-like proteins extracted from publicly available bacterial genomes, we identify 125 putative novel *mcr*-like genes (representing 13 clusters at an 80% AA identity threshold), which may be co-harbored on plasmids with other antimicrobial resistance (AMR) genes. Overall, the results presented here provide insight into the evolution and diversity of *mcr* and *ipet* genes.

## 2 Materials and methods

### 2.1 Acquisition of MCR and MCR-like amino acid sequences

A representative amino acid (AA) sequence from each published MCR family (i.e., at the time of the search, MCR-1 to -9, accessed April 23, 2019; **Supplementary Table S1**) was queried against the National Center for Biotechnology Information (NCBI) non-redundant (nr) protein database using the protein BLAST (BLASTP) webserver (https://blast.ncbi.nlm.nih.gov/Blast.cgi?PAGE=Proteins; accessed April 23-24, 2019) (Johnson et al., 2008). BLASTP default settings were used for all parameters except max target sequences, which was raised to 5,000. BLASTP hits corresponding to proteins with high query coverage (i.e., ≥90% of the length of the original MCR query) were maintained in subsequent steps (*n* = 41,270 of 45,001 total hits). Because MCR families can share low degrees of AA identity (e.g., < 40%) (Partridge et al., 2018), no additional sequence identity thresholds were employed so that potentially novel, remote MCR homologs could be identified. From all 41,270 BLASTP hits, the union of all nr protein accession numbers was taken, yielding a total of 9,866 nr protein accession numbers associated with MCR and MCR-like proteins. The AA sequences of all 9,866 MCR and MCR-like proteins, as well as all associated NCBI Identical Protein Group (IPG) accession numbers, were downloaded using the rentrez package version 1.2.1 (Winter, 2017) in R version 3.5.3 (R Core Team, 2021). Finally, known MCR families are similar in terms of sequence length (e.g., the representatives of MCR-1 to -9 listed in **Supplementary Table S1** ranged from 538-565 AA in length, with an average/median length of 545/541 AA). We thus removed BLASTP hits that represented extreme outliers based on sequence length as follows: the median absolute deviation of the lengths of the AA sequences of all MCR and MCR-like proteins was calculated, and AA sequences of MCR-like proteins falling outside the range of sequence lengths encompassed by 15 times the median absolute deviation were removed (with 30 proteins removed by this step).

Overall, this initial BLASTP search yielded a total of 9,836 MCR and MCR-like AA sequences, which were used in subsequent steps (**Supplementary Table S2**). While this search did not specifically query MCR families and alleles described after the access date (i.e., April 23, 2019), the selected BLASTP parameters allowed us to detect MCR families and alleles, which were in NCBI’s nr database but were not yet discovered and published. For example, MCR-10 was not specifically queried in the initial BLASTP search (**Supplementary Table S1**), as it had not yet been discovered (Wang et al., 2020). However, the approach described above identified MCR-10.1 in NCBI’s nr database prior to its discovery, and MCR-10.1 was thus included among the 9,836 MCR and MCR-like proteins used in subsequent steps (NCBI Protein Accession WP_023332837.1; **Supplementary Table S2**).

### 2.2 Acquisition and characterization of genomes harboring MCR-like proteins

NCBI RefSeq Assembly accession numbers for all genomes associated with ≥1 MCR or MCR-like protein in NCBI’s IPG database were acquired via rentrez (see section “Acquisition of MCR and MCR-like amino acid sequences” above). Latest assembly versions for all RefSeq genomes were then downloaded via NCBI’s FTP site (*n* = 59,129 total genomes; accessed May 19, 2020). To confirm that ≥1 MCR or MCR-like protein could be detected in each genome, the BLASTX algorithm implemented in DIAMOND version 0.9.13.114 (Buchfink et al., 2015) was used to perform a translated search of each genome against AA sequences of all 9,836 MCR and MCR-like proteins identified as described above, plus the AA sequences of all 53 known MCR alleles available in ResFinder at the time (so that proteins that most closely resembled a known MCR allele would be labeled as such; https://cge.cbs.dtu.dk/services/ResFinder/, see section “Acquisition of MCR and MCR-like amino acid sequences” above) (Zankari et al., 2012). The following DIAMOND BLASTX parameters were used to confirm the presence of MCR and/or MCR-like proteins: a maximum E-value threshold of 1e-5 (-e 0.00001), a minimum subject coverage threshold of 90% (–subject-cover 90), a minimum percent AA identity of 90% (–id 90). This resulted in a total of 69,814 confirmed hits of MCR and MCR-like proteins in 59,121 RefSeq genomes, which were used in subsequent steps (**Supplementary Table S2**).

To assign each genome to a species using a standardized taxonomic framework, all 59,121 genomes were assigned to a marker-gene based operational taxonomic unit (mOTU) using classify-genomes (accessed June 3, 2020; https://github.com/AlessioMilanese/classify-genomes) and version 2.5 of the mOTUs taxonomy (Milanese et al., 2019) (**Supplementary Table S2**). Antimicrobial resistance (AMR) genes were detected in each genome via the nucleotide BLAST-based approach implemented in ABRicate version 0.9.8 (https://github.com/tseemann/abricate), using the NCBI National Database of Antibiotic Resistant Organisms (NDARO; accessed April 19, 2020) (Feldgarden et al., 2019). ABRicate was additionally used to detect plasmid replicons present in the PlasmidFinder database (Carattoli et al., 2014) in each genome (accessed April 19, 2020). AMR genes and plasmid replicons were considered to be present in a genome using minimum nucleotide identity and coverage thresholds of 80% each (**Supplementary Table S2**). PlasFlow version 1.1.0 (Krawczyk et al., 2018) was additionally used to predict whether contigs were plasmid-associated or chromosomal in origin (using default settings; **Supplementary Table S2**).

### 2.3 Identification of putative novel *mcr*-like genes

Of the 69,814 MCR and MCR-like proteins identified in 59,121 genomes from NCBI’s RefSeq database (see section “Acquisition and characterization of genomes harboring MCR-like proteins” above), we identified 321 MCR-like proteins, which were located on the same contig as (i) ≥1 plasmid replicon (detected via ABRicate and the PlasmidFinder database) and (ii) ≥1 additional AMR gene (detected via ABRicate and the NDARO), with contigs harboring previously described *mcr* alleles excluded (see section “Acquisition and characterization of genomes harboring MCR-like proteins” above for details regarding plasmid replicon and AMR gene detection; **Supplementary Table S2**). These criteria were used to identify and prioritize putative novel MCR-like proteins, as (i) the presence of a plasmid replicon on the same contig as an MCR-like protein indicated that the MCR-like protein and plasmid replicon were likely to be in close proximity (thus predicting that the gene was on a plasmid, rather than a chromosome); and (ii) the presence of other AMR genes on the same contig as an MCR-like protein indicated that the MCR-like protein may have been harbored on a plasmid that was potentially associated with multidrug-resistance. These 321 MCR-like proteins represented 125 unique nucleotide sequences, which we will refer to hereafter as “putative novel *mcr*-like genes” and their products as “putative novel MCR-like proteins” (**Supplementary Table S3**).

### 2.4 Acquisition of chromosomally encoded intrinsic lipid modification PET genes

Closed representative genome sequences of 147 reference species known to harbor *mcr* genes were downloaded from the NCBI RefSeq Assembly database (accessed March 12, 2021). These included Enterobacteriaceae (NCBI txid543), *Proteus* (NCBI txid583), *Aeromonas* (NCBI txid642), and *Moraxella* (NCBI txid475) species (**Supplementary Table S4**). Assembled genomes were imported into Geneious version 2019.2.3 (Biomatters, Auckland, New Zealand), and sequences of complete closed chromosomes were extracted for subsequent analysis. AA sequences of *E. coli* str. K-12 substr. MG1655 CptA (NCBI Protein Accession WP_000556306.1), EptA (NCBI Protein Accession WP_000919792.1), and EptB (NCBI Protein Accession WP_001269197.1) were queried against the closed chromosome sequences using translated nucleotide BLAST (tBLASTN, as implemented in Geneious version 2019.2.3). A total of 237 unique hits that showed ≥90% query coverage were selected to represent chromosomally encoded intrinsic lipid modification PET genes (**Supplementary Table S5**). We will collectively refer to *eptA*, *eptB*, and *cptA* intrinsic lipid modification PET-encoding genes as *ipet* genes and their products as i-PET enzymes or proteins.

### 2.5 Construction of maximum likelihood phylogeny of *mcr* and *mcr*-like genes

Maximum likelihood (ML) phylogenies were inferred from the nucleotide sequences of (i) all *mcr* alleles available in NCBI’s NDARO (*n* = 98, accessed March 12, 2021; **Supplementary Table S6**), (ii) putative novel *mcr*-like genes identified in this study (*n* = 125; see section “Identification of putative novel *mcr*-like genes” above) and/or (iii) *ipet* (*n* = 237) coding sequences (see section “Acquisition of chromosomally encoded intrinsic lipid modification PET genes” above; **Supplementary Table S7**). Back-translated nucleotide multiple sequence alignments (NT_btn_-MSA) were constructed using MUSCLE (Edgar, 2004) with the default settings in Geneious version 2019.2.3. The resulting NT_btn_-MSAs were used to construct ML phylogenies with 100 bootstrap replicates via RAxML, using the GTRGAMMA nucleotide substitution model and default settings (RAxML GUI version 2.0.7 and RAxML version 8.2.12) (Stamatakis, 2014). The resulting trees were visualized and edited using iTOL version 6.5 (https://itol.embl.de/) (Letunic and Bork, 2007).

### 2.6 Clustering of PET amino acid sequences

AA sequences of the MCR, putative novel MCR-like, and i-PET proteins (*n* = 98, 125, and 237 proteins, respectively; **Supplementary Table S7**) were clustered into families using CD-HIT version 4.8.1 (Li and Godzik, 2006; Fu et al., 2012) with an 80% AA sequence identity threshold (-c 0.8) and a word length of 5 (-n 5; i.e., the value suggested by CD-HIT for the given AA sequence identity threshold). CD-HIT clusters that did not contain a known MCR allele at the 80% AA sequence identity threshold were treated as putative novel MCR families, as 80% AA identity represents a conservative threshold for MCR family delineation (e.g., at this threshold, MCR-1, MCR-2, and MCR-6 clustered together and would be considered to be members of the same MCR family) (Partridge et al., 2018).

### 2.7 Descriptive sequence analysis

The number of polymorphic sites, nucleotide diversity per site, average pairwise nucleotide differences per sequence, number of synonymous substitutions (S) and non-synonymous substitutions (N), and the *dN/dS* ratio for each NT_btn_-MSA were calculated using DnaSP version 6.12.03 (**Supplementary Table S7**; see section “Construction of maximum likelihood phylogeny of *mcr* and *mcr*-like genes” above) (Rozas et al., 2017).

### 2.8 Homologous recombination detection

The Recombination Detection Program (RDP5) was used to detect homologous recombination events within the NT_btn_-MSA of *mcr*, putative novel *mcr*-like, and *ipet* genes (*n* = 98, 125, and 237 genes, respectively; **Supplementary Table S7**) (Martin et al., 2021). Seven homologous recombination detection methods (RDP, BOOTSCAN, GENECON V, MAXCHI, CHIMAERA, SISCAN, and 3Seq) were used to perform a full exploratory recombination analysis using the default settings in RDP5. Only recombination events detected by at least three methods were selected for further analysis to reduce false positives. Recombination events with similar breakpoints were merged into a single event, and the overall significance of the recombination evidence for each event was evaluated via the Pairwise Homoplasy Index (PHI) test with default settings in RDP5.

### 2.9 Positive selection analysis

The Datamonkey server (https://www.datamonkey.org/) (Weaver et al., 2018) was used to identify *mcr* residues that evolved under positive selection. The “hyphy cln” command within HyPhy version 2.5.32(MP) (Kosakovsky Pond et al., 2020) was used to remove stop codons within the NT_btn_-MSA of the 98 known *mcr* alleles (see section “Construction of maximum likelihood phylogeny of *mcr* and *mcr*-like genes” above; **Supplementary Table S6**). The resulting cleaned alignment was supplied as input to the command line implementation of GARD (Kosakovsky Pond et al., 2006; Weaver et al., 2018; Kosakovsky Pond et al., 2020), which was used to detect recombination breakpoints within the alignment using default settings. GARD partitioned the dataset into fragments of 1-1,204 bp and 1,205-1,779 bp.

The resulting partitioned dataset produced by GARD (with suffix ".best.gard") was supplied as input to the following (both accessed January 27, 2022): (i) FUBAR (Fast, Unconstrained Bayesian AppRoximation; https://www.datamonkey.org/fubar), which uses a Bayesian approach to infer non-synonymous (*dN*) and synonymous (*dS*) substitution rates on a per-site basis and assumes constant selection pressure for each site along the entire phylogeny (Murrell et al., 2013); (ii) MEME (mixed-effect model evolution-based positive selection analysis; https://www.datamonkey.org/meme), which tests the hypothesis that individual sites have been subjected to episodic positive selection (Murrell et al., 2012). For both FUBAR and MEME, the universal genetic code option was selected; for FUBAR, additional parameters under “advanced options” were set to their default values.

### 2.10 *In silico* structural modeling and construction of sequence logos

Structural modeling of MCR-1 was done using the Phyre2 server (accessed June 20, 2021) (Kelley et al., 2015) based on *Neisseria meningitidis* lipooligosaccharide phosphoethanolamine transferase EptA (Anandan et al., 2017). The protein structure was viewed and annotated using UCSF ChimeraX version 1.3 (Pettersen et al., 2021). Residues predicted to have evolved under positive selection were mapped on the MCR-1 protein 3D structural model using UCSF ChimeraX (Pettersen et al., 2021).

An NT_btn_-MSA and encoded AA-based MSA of the 98 known *mcr* alleles were constructed using MUSCLE (Edgar, 2004) with default settings in Geneious version 2019.2.3. The ESPript 3 server version 3.0.8 was used to align MCR-1 secondary structure elements onto the AA MSA of the 98 known MCR alleles (Robert and Gouet, 2014). Residues under negative or positive selection were mapped onto the AA MSA of the 98 known MCR alleles in Geneious version 2019.2.3. The 19 AA residues and codons (57 bases) identified by MEME were extracted from the corresponding MSA across the 98 *mcr* alleles in Geneious and saved as separate MSA FASTA files. The resulting MSA FASTA files were used to construct AA and nucleotide graphical representation logos via the WebLogo3 server (accessed May 26, 2022) (Crooks et al., 2004).

An AA-based MSA of MCR, putative novel MCR-like, and i-PET proteins (*n* = 98, 125, and 237 proteins, respectively; **Supplementary Table S7**) was constructed using MUSCLE with default settings in Geneious version 2019.2.3 (Edgar, 2004). The ESPript 3 server version 3.0.8 was used to align EptA secondary structure elements onto the MSA (Robert and Gouet, 2014). The resulting MSA was used to visually identify cysteine residues involved in disulfide bond formation across the different proteins based on the experimentally identified disulfide bonds in the EptA structure (Anandan et al., 2017) and site-directed mutagenesis analyses of the corresponding sites in CptA (Zhao et al., 2019).

### 2.11 Characterization of the genomic context of PET-encoding genes

To characterize the genomic context surrounding different PET-encoding genes, genomic regions 5 Kb upstream and downstream of all PET-encoding genes were extracted from each PET-harboring genome (i.e., all genomes in which MCR and MCR-like proteins were detected, and all i-PET-harboring genomes used in this study; **Supplementary Tables S2**, **S4**, respectively). NCBI Prokaryotic Genome Annotation Pipeline (PGAP) annotations (Tatusova et al., 2016) were used to identify genes directly upstream and downstream of PET-encoding genes (i.e., not separated from PET-encoding genes by another ORF). Sequences were visually examined, and genes were considered to be in the same operon if they were located within approximately < 20 nucleotides and had no apparent promoter-like sequence in this intergenic region.

### 2.12 Collection of phenotypic colistin resistance data

Colistin minimum inhibitory concentration (MIC) data were collected from the literature (**Supplementary Table S8**) (Di Pilato et al., 2016; Liu et al., 2016; Xavier et al., 2016; Abuoun et al., 2017; Borowiak et al., 2017; Carattoli et al., 2017; Ling et al., 2017; Liu et al., 2017; Lu et al., 2017; Poirel et al., 2017; Tijet et al., 2017; Yang et al., 2017; Yin et al., 2017; Zhao et al., 2017; Alba et al., 2018; Carattoli et al., 2018; Chavda et al., 2018; Dortet et al., 2018; Duggett et al., 2018; Eichhorn et al., 2018; Fernandes et al., 2018; Garcia-Graells et al., 2018; Hammerl et al., 2018; Kieffer et al., 2018; Li et al., 2018; Liu et al., 2018; Poirel et al., 2018; Rebelo et al., 2018; Shen et al., 2018; Teo et al., 2018; Wang et al., 2018b; Wang et al., 2018c; Wise et al., 2018; Xiang et al., 2018; Xu et al., 2018b; Xu et al., 2018c; Yang et al., 2018; Abdul Momin et al., 2019; Bitar et al., 2019; Chavda et al., 2019; Cui et al., 2019; Deshpande et al., 2019; Hadjadj et al., 2019; Li et al., 2019; Long et al., 2019; Wang et al., 2019; Yang et al., 2019; Yuan et al., 2019; Zhang et al., 2019; Zheng et al., 2019; Bitar et al., 2020; Cha et al., 2020; Fan et al., 2020; Garcia-Menino et al., 2020; Hatrongjit et al., 2020; Lei et al., 2020; Martins-Sorenson et al., 2020; Neumann et al., 2020; Ngbede et al., 2020; Wang et al., 2020; Anyanwu et al., 2021; Hu et al., 2021; Khanawapee et al., 2021; Leangapichart et al., 2021; Snyman et al., 2021; Stosic et al., 2021; Uddin et al., 2021; Yu et al., 2021). A search of NCBI’s PubMed database (https://pubmed.ncbi.nlm.nih.gov/) was conducted on August 13, 2021, using keywords [*mcr* and colistin resistance]. The search results were exported to an EndNote library (EndNote 20, Clarivate, Philadelphia, USA). A similar search was performed on the Web of Science Core Collection database (Clarivate, Philadelphia, USA), and the search results were used to amend the EndNote library. Duplicate entries in the EndNote library were deleted, and full-text PDF files were downloaded using EndNote “Find Full Text”. When needed, **Supplementary Materials** were downloaded and incorporated into the PDF file.

The EndNote library was searched for each known *mcr* allele using the allele designation (e.g., *mcr-3.3*) with the “Any Field + PDF with Notes” search criteria. The search results were sorted by date to identify the primary publication(s) describing the allele for the first time. The full-text articles were visually scanned to identify the publication(s) describing (i) the identification of the allele and (ii) the functional characterization of colistin resistance. The “Materials and Methods” and “Results” sections that described colistin resistance phenotypic data were examined carefully, and colistin MIC data were collected. We opted to use MIC values determined by the broth microdilution method, which is recommended by the European Committee on Antimicrobial Susceptibility Testing (EUCAST) (Jayol et al., 2018). We excluded MIC data determined by agar dilution and gradient agar disk diffusion (colistin gradient strip zone of inhibition) methods, which have been suggested to be less accurate in determining colistin resistance levels when compared to the broth microdilution method (Chew et al., 2017; Garcia-Menino et al., 2020; Yusuf et al., 2020). If no data were identified in the EndNote library for specific *mcr* alleles, we performed an additional search using Google Scholar, using the given allele’s designation.

Colistin MIC values reported for native *mcr*-harboring strains (**Supplementary Table S8**) were base-2 logarithm-transformed using the log2 function in R and displayed in a heatmap using iTOL version 6. An ML phylogeny of species reported for the native *mcr*-harboring strains was additionally supplied to iTOL. Briefly, a type strain and/or NCBI species reference genome was downloaded from NCBI’s RefSeq Assembly database for each *mcr*-harboring native strain species (*n* = 18 total species, with one genome selected per species; **Supplementary Table S8**). The “classify_wf” workflow implemented in the Genome Taxonomy Database Toolkit (GTDB-Tk) version 2.1.0 was used to construct an AA MSA of all 18 genomes, as well as to confirm species assignments reported in NCBI (using version R207_v2 of GTDB) (Chaumeil et al., 2019; Parks et al., 2022). The AA MSA produced by GTDB-Tk (i.e., the file named “gtdbtk.bac120.user_msa.fasta”) was supplied as input to IQ-TREE version 1.5.4 (Nguyen et al., 2015), which was used to construct the ML phylogeny, using (i) the optimal AA substitution model selected via Bayesian Information Criteria values produced via ModelFinder (i.e., the “LG+F+R3” model) (Yang, 1995; Le and Gascuel, 2008; Soubrier et al., 2012; Kalyaanamoorthy et al., 2017); and (ii) one thousand replicates of the ultrafast bootstrap approximation (Minh et al., 2013).

## 3 Results

### 3.1 MCR-like proteins are distributed across a wide range of Gram-negative bacterial taxa

A protein BLAST (BLASTP)-based search for MCR and MCR-like proteins in NCBI’s nr and IPG databases showed that MCR- and MCR-like proteins were distributed across a wide range of Gram-negative bacterial taxa (**Figure 1**). More than 69,000 BLASTP hits against known MCR families were detected in whole-genome sequencing (WGS) data spanning 256 bacterial genera, including *Escherichia*, *Shigella*, *Salmonella*, *Klebsiella*, *Aeromonas*, *Moraxella*, *Enterobacter*, *Xanthomonas*, and *Pseudomonas* spp. (per the mOTUs taxonomy; **Supplementary Table S2**). Among the 69,814 total BLASTP hits to MCR and MCR-like proteins identified here, 15,321 (21.9%) corresponded to proteins annotated as EptA (i.e., the associated protein was annotated in NCBI with the term “EptA” or “*eptA*”; **Supplementary Table S2**). Mobilization is a hallmark of *mcr*, and insertion sequence (IS) elements have been found to flank some *mcr* alleles; thus, we also searched for transposon-related elements (e.g., IS elements or transposase genes) within the 5 Kb flanking regions of the 69,814 *mcr* and *mcr*-like genes identified here (**Supplementary Table S2**). Overall, we found that 1,097 of the 69,814 *mcr* and *mcr*-like genes possessed transposon-related elements within their 5 Kb flanking regions (based on NCBI Prokaryotic Genome Annotation Pipeline [PGAP] annotations within these regions, accessed May 19-21, 2020; **Supplementary Table S2**) (Tatusova et al., 2016). It has been reported that multiple *mcr* alleles have lost one or both IS elements (Snesrud et al., 2017; Snesrud et al., 2018; Wang et al., 2018a; El-Sayed Ahmed et al., 2020). Thus, we did not include the presence of IS elements as a criterium for the identification of *mcr* and putative novel *mcr*-like genes in this study.

As *mcr* genes are typically plasmid-borne, while intrinsic lipid modification PET-encoding genes (*ipet* genes) are typically chromosomally encoded, we hypothesized that *mcr*-like genes detected on the same contig as a plasmid replicon and other antimicrobial resistance (AMR) genes were more likely to encode PET associated with colistin resistance. Initially, we identified 321 BLASTP hits to MCR proteins, which met these criteria (defined here as “putative novel MCR-like proteins” and their encoding genes designated as “putative novel *mcr*-like genes”; **Figure 1B** and **Supplementary Table S3**). Sequence similarities showed that the 321 putative novel *mcr*-like genes represented 125 unique nucleotide sequences, which were selected for further analyses (**Figure 1B** and **Supplementary Tables S3**, **S7**). To further analyze these genes, we created a back-translated nucleotide multiple sequence alignment (NT_btn_-MSA) of the 125 unique, putative novel *mcr-*like genes identified here, along with 98 known *mcr* alleles (**Supplementary Tables S3**, **S6**). These 223 sequences showed an average pairwise nucleotide diversity per site (p) of 0.42675, with an average number of pairwise nucleotide differences per sequence (k) of 623.052 over 1,776 sites. The relatively high p value observed here was not surprising, as it has been shown previously that *mcr* families encompass an extensive degree of genetic diversity (Khedher et al., 2020); this is also evident within an NT_btn_-MSA constructed using the 98 known *mcr* alleles alone (i.e., p = 0.3614 and k = 553.669 over 1,776 sites; **Supplementary Table S6**).

**Figure 2:**
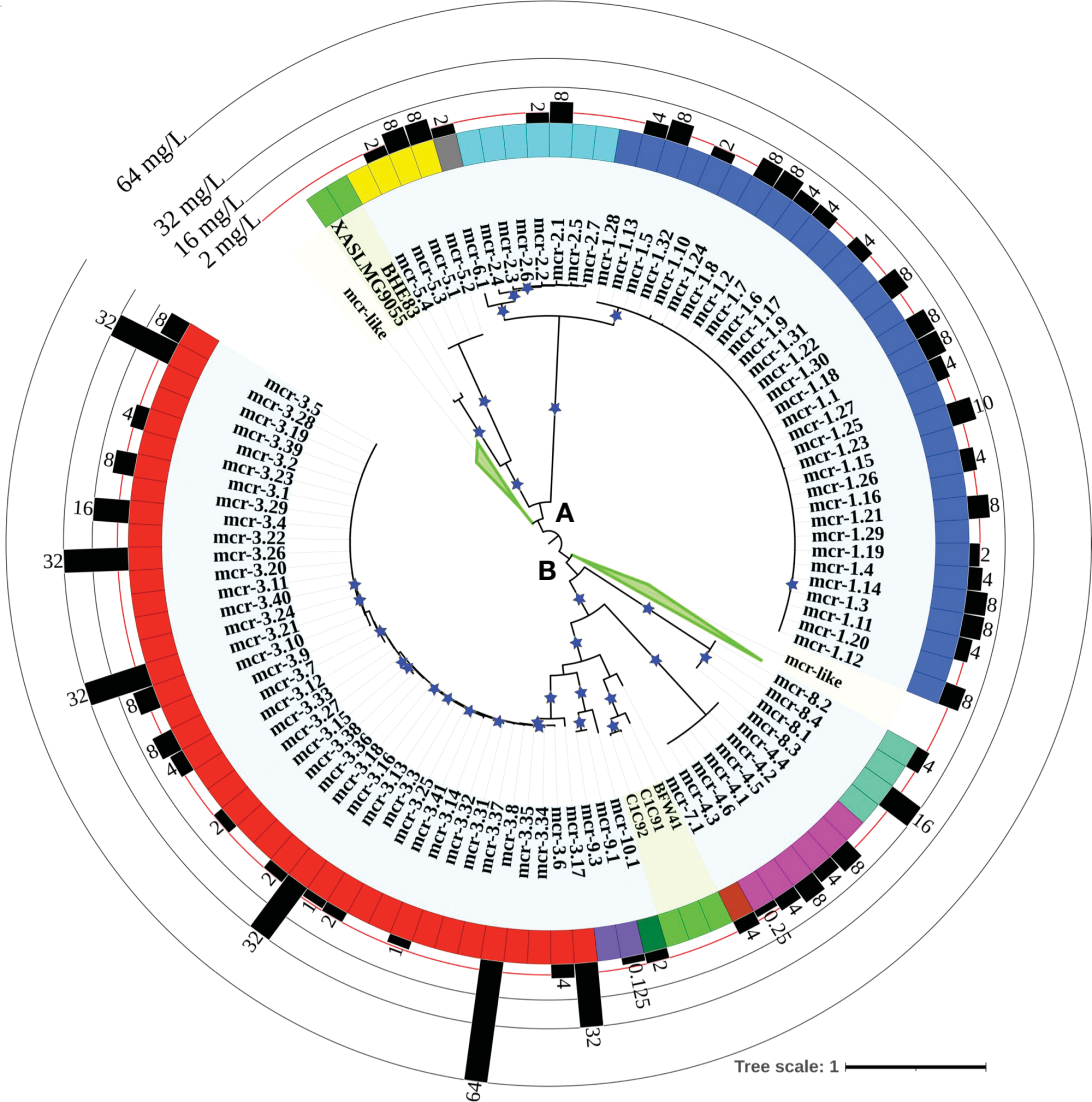
Maximum likelihood (ML) phylogeny inferred from a nucleotide back-translation-based multiple sequence alignment (NT_btn_-MSA) of (i) 98 *mcr* alleles and (ii) 125 unique sequences of *mcr*-like genes located on the same contig as ≥1 plasmid replicon and ≥1 other antimicrobial resistance (AMR) gene (i.e., “putative novel *mcr*-like genes”). Sequences were aligned using MUSCLE. The ML phylogeny was constructed with RAxML, using the GTRGAMMA nucleotide substitution model and 100 bootstrap replicates. The tree was edited using the iTOL web server (https://itol.embl.de/) and rooted at the midpoint, with branch lengths reported in substitutions per site. Branches with bootstrap values ≥70% are denoted by blue stars. Clades exclusively composed of putative novel *mcr*-like genes were collapsed for clarity (green branches; see **Supplementary Figure S1** for a fully expanded tree). Tip label shading corresponds to known *mcr* alleles (light blue) and putative novel *mcr*-like genes identified in this study (light green). The colors of the inner ring represent different *mcr* families (*mcr-1* to *-10*). The outer graph represents the maximum reported colistin minimum inhibitory concentration (MIC) values of native strains harboring different *mcr* alleles. The colistin breakpoint established by the Clinical and Laboratory Standards Institute (CLSI) is 2 mg/L (red line). Isolates with colistin MIC ≥2 mg/L are considered colistin-resistant. Colistin MIC values were compiled from different *mcr*-harboring Gram-negative species as listed in **Supplementary Table S8**; metadata associated with each gene can be found in **Supplementary Table S6**.

A maximum likelihood (ML) phylogeny inferred from the NT_btn_-MSA of known *mcr* alleles and putative novel *mcr*-like genes showed a robust separation of clades and subclades representing different *mcr* families (**Figure 2** and **Supplementary Figure S1**). Specifically, *mcr* alleles clustered into two distinct phylogenetic lineages: (i) lineage A included alleles belonging to the *mcr-1*, *mcr-2*, *mcr-5*, and *mcr-6* families, and (ii) lineage B included all remaining *mcr* alleles (**Figure 2** and **Supplementary Figure S1**). Interestingly, both clades contained putative novel *mcr*-like genes, with 21 and 103 putative novel *mcr*-like genes clustering into lineage A and B, respectively (**Figure 2** and **Supplementary Figure S1**). While some putative novel *mcr*-like genes clustered with known *mcr* families (i.e., three genes clustered with *mcr-7.1* and two genes clustered with *mcr-5*; **Supplementary Figure S1**), the majority of novel *mcr*-like genes grouped into two large phylogenetic clades that were distinct from known *mcr* families (**Figure 2** and **Supplementary Figure S1**).

### 3.2 Some *mcr* alleles are more closely related to *eptA* than to other *mcr* alleles

Consistent with the wide range of genetic diversity observed among nucleotide sequences of *mcr* families, AA sequence similarities among MCR families also varied widely, with similarities ranging from 59.3% to 100% and identities ranging from 29.7% to 99.8% (**Supplementary Tables S9**, **S10**). To gain further insight into MCR diversity in the context of intrinsic lipid modification PET (i-PET), we identified and aggregated AA and nucleotide sequences associated with 237 chromosomally encoded i-PET proteins (i.e., EptA, EptB, and CptA; **Supplementary Table S5**). These i-PET proteins were extracted from 147 genomes of genera, which had been reported to harbor *mcr* (e.g., *Aeromonas*, *Citrobacter*, *Cronobacter*, *Enterobacter*, *Escherichia*, *Klebsiella*, *Moraxella*, *Proteus*, and *Salmonella* spp.; **Supplementary Tables S4**, **S5**) (Nang et al., 2019). The nucleotide and AA sequences of the 237 chromosomally encoded i-PET proteins identified here were compared to those of the 98 known MCR alleles, as well as the sequences of the 125 putative novel MCR-like proteins identified here (*n* = 460 total PET; **Supplementary Table S7**). Based on nucleotide sequences, all 460 PET-encoding genes queried here displayed extensive sequence diversity (i.e., p = 0.50571 and k = 450.085; **Supplementary Table S7**).

Based on AA sequences, MCR alleles shared a relatively low degree of AA similarity with EptB and CptA, ranging from 46% to 58% and 42% to 49%, respectively (**Supplementary Tables S9** and **S10**). Notably, however, MCR alleles differed in terms of the degree of AA similarity they shared with EptA: overall, MCR allele AA similarities to EptA ranged from 61% to 76%, while some MCR alleles shared a higher degree of AA similarity to EptA than to other MCR alleles (**Supplementary Tables S9**, **S10**). For example, MCR-8.1 showed average similarities of 72.8 ± 1.1% to EptA and 70 ± 7% to other MCR alleles (**Supplementary Tables S9** and **S10**). Conversely, MCR-1.1 showed average similarities of 64.5 ± 1.5% to EptA and 76.1 ± 18.1% to other MCR alleles (**Supplementary Tables S9** and **S10**). This suggests that sequence similarity alone cannot sufficiently discriminate canonic EptA from MCR.

### 3.3 Colistin resistance and intrinsic lipid modification PET proteins represent distinct clades, which show some associations with PET functionality

**Figure 3:**
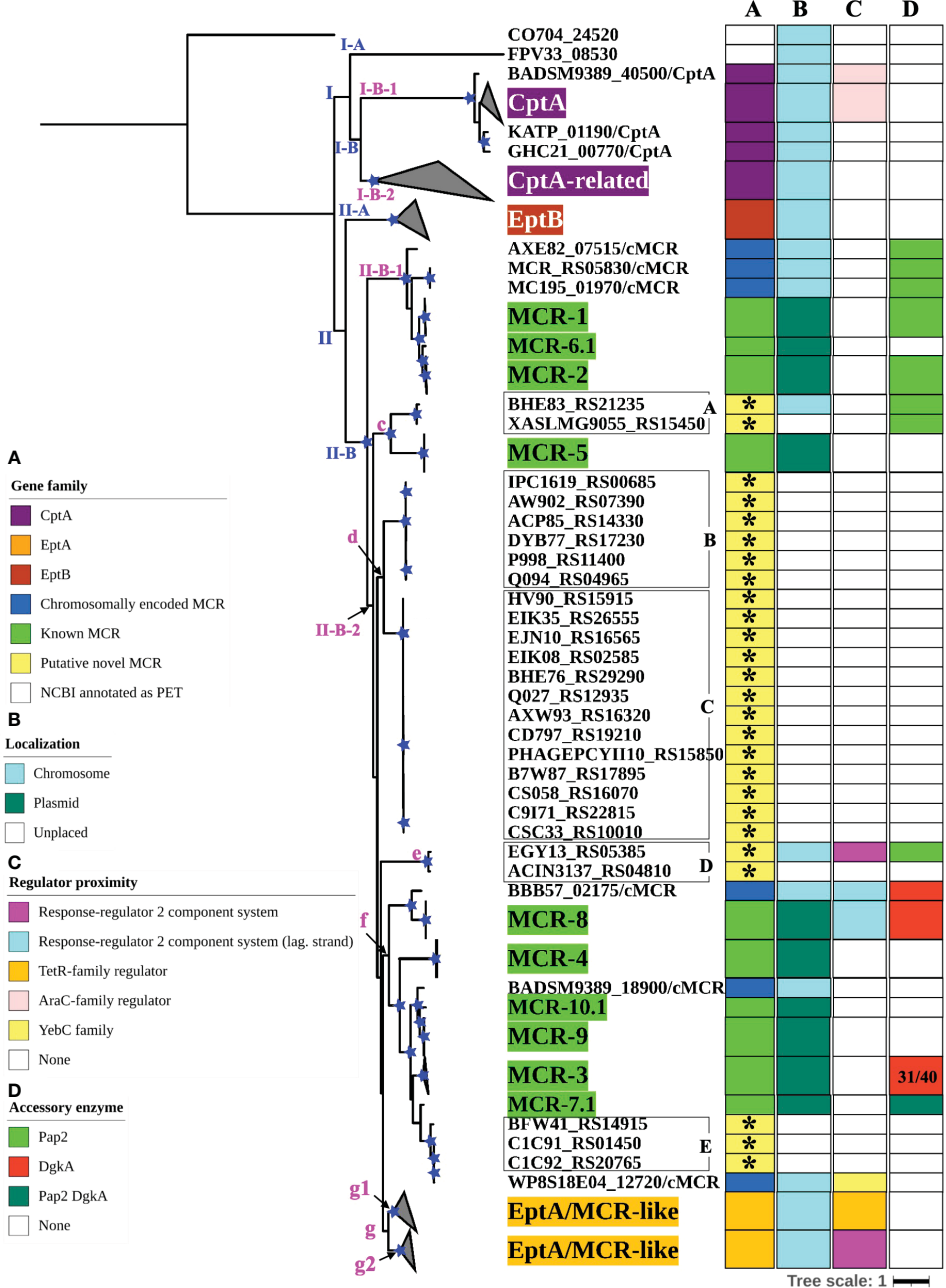
Maximum likelihood (ML) phylogeny inferred from a nucleotide back-translation-based multiple sequence alignment (NT_btn_-MSA) of **(i)** 98 *mcr* alleles, **(ii)** 125 unique sequences of *mcr*-like genes located on the same contig as ≥1 plasmid replicon and ≥1 other antimicrobial resistance (AMR) gene (i.e., “putative novel *mcr*-like genes”), and **(iii)** 237 chromosomal phosphoethanolamine transferase (*ipet*) genes. Sequences were aligned using MUSCLE. The ML phylogeny was constructed with RAxML, using the GTRGAMMA nucleotide substitution model and 100 bootstrap replicates. The tree was edited using the iTOL web server (https://itol.embl.de/) and rooted at the midpoint, with branch lengths reported in substitutions per site. Branches with bootstrap values ≥70% are denoted by blue stars. Linages and clades described in the main text are numbered in blue, while subclades and clusters are numbered in magenta. Clades exclusively composed of genes from the same family were collapsed and color-coded, as shown in the left color legend key (see **Supplementary Figure S2** for a fully expanded tree). Five PET families clustered based on an 80% AA identity threshold representing 26 putative novel *mcr* genes are identified by black boxes around the taxa names with capital letters A to E and asterisks in color strip **(A)**. Color-coded regions in strip **(A)** denote *mcr* families, putative novel *mcr*-like genes, and *eptA, eptB*, and *cptA* homologues. Color-coded regions in strip **(B)** denote gene localization as chromosomally encoded (cyan), plasmid-encoded (green), or unplaced (white); localization was assigned based on the first gene reported in the literature (note that for some gene families, e.g., *mcr-3*, chromosomally encoded genes have also been reported). Color-coded regions in strip **(C)** represent the regulatory system juxtaposing phosphoethanolamine transferase (PET)-encoding genes: magenta regions represent genes contained within the same operon as a two-component response sensor-regulatory system; cyan regions represent genes located divergent of a two-component response sensor-regulatory system; orange regions represent genes located adjacent to a TetR-type regulator; pink regions represent genes located adjacent to an AraC-type regulator; yellow region represents gene located adjacent to a YebC-type regulator; white regions represent genes represented by single-gene operons with no upstream or downstream regulatory protein. Color-coded regions in strip **(D)** represent PET-encoding genes localized adjacent to accessory enzyme-encoding genes, including *pap2* encoding lipid A phosphatase (light green), *dgkA* encoding diacylglycerol kinase (red), or both *pap2* and *dgkA* genes (dark green); white regions represent the absence of accessory enzyme-encoding genes upstream or downstream of PET-encoding genes.

To further characterize the diversity and evolution of PET-encoding genes, we constructed an ML phylogeny using the nucleotide sequences of the 98 known *mcr*, 125 putative novel *mcr*-like, and 237 *ipet* genes identified here (representing a total of 460 PET-encoding nucleotide sequences; monophyletic groups within this phylogeny will be referred to as “lineages”, followed by “clades”, “subclades”, and “clusters”, **Supplementary Table S7**). The phylogeny of these 460 PET-encoding genes included one singleton gene, as well as (i) lineage I (105 taxa), which included *cptA* genes, and (ii) lineage II (354 taxa), which included *eptA*, *eptB*, *mcr*, and all 125 putative novel *mcr*-like genes (**Figure 3** and **Supplementary Figure S2**). Lineage I included clades I-A and I-B, with two large I-B subclades (subclade I-B-1 and I-B-2; **Figure 3** and **Supplementary Figure S2**). Subclade I-B-1 represented *bona fide cptA*, based on sequence similarity to experimentally confirmed *cptA* genes (Tamayo et al., 2005a). Subclade I-B-2, on the other hand, represented 71 chromosomally encoded *ipet* genes, but did not include any experimentally confirmed *cptA* genes (**Figure 3** and **Supplementary Figure S2**). The fact that subclade I-B-2 clustered with the *cptA*-containing subclade I-B-1 may, however, indicate that genes in subclade I-B-2 encode enzymes with acceptor substrate specificities similar to, but not identical to, the substrate specificity of CptA. Within lineage II, *eptA* and *eptB* clustered into distinct phylogenetic groups (clade II-A and cluster II-B-2g, respectively; **Figure 3** and **Supplementary Figure S2**). Overall, this suggests clustering of PET-encoding genes is correlated with the enzymes’ acceptor substrate specificities, consistent with previous reports (Harper et al., 2017) (**Figure 3** and **Supplementary Figure S2**).

Clade II-B represented subclades II-B-1 and II-B-2; alleles belonging to the *mcr-1*, *mcr-2*, and *mcr-6* families clustered into subclade II-B-1, while alleles belonging to the remaining *mcr* families clustered into subclade II-B-2 (along with the *eptA* genes, which represent cluster II-B-2g; **Figure 3** and **Supplementary Figure S2**). Overall clustering was consistent between this phylogeny (**Figure 3**) and the phylogeny based only on *mcr* and *mcr*-like genes (**Figure 2**), expect for the placement of the *mcr-5* family and two closely related genes (i.e., gene loci BHE83_RS21235 and XASLMG9055_RS15450), which were placed with *mcr-1*, *mcr-2* and *mcr-6* in **Figure 2** and with *mcr-3*, *mcr-4*, *mcr-7*, *mcr-8*, *mcr-9*, and *mcr-10* in **Figure 3** (consistent with the fact that the *mcr-5* branch did not receive strong bootstrap support in either phylogeny; **Figures 2**, **3**). Notably, all 125 putative novel *mcr*-like genes were grouped into subclade II-B-2; 99 of these genes clustered with *eptA* genes (cluster II-B-2g) rather than with known *mcr* alleles (**Figure 3**), even though these 99 genes were co-localized on a contig with a plasmid replicon and additional AMR gene(s) (**Supplementary Tables S2**, **S3**). The remaining 26 putative novel *mcr-*like genes clustered with known *mcr* alleles in the ML phylogeny, including (i) two and three genes that clustered with *mcr-5* and *mcr-7* (clusters II-B-2c and II-B-2f; **Figure 3**), and (ii) 21 putative novel *mcr-*like genes that formed two distinct phylogenetic clusters (groups II-B-2-d and II-B-2e; **Figure 3**). These data suggest that these 26 putative novel *mcr*-like genes are likely to be previously unidentified *mcr* genes.

Our phylogeny also provided further evidence that some *mcr* families and alleles (i.e., *mcr-3*, *mcr-4*, *mcr-5*, *mcr-8*, and *mcr-7.1*) are more closely related to *eptA* than to other *mcr* alleles (i.e., *mcr-1*, *mcr-2*, and *mcr-6.1*; **Figure 3** and **Supplementary Figure S2**) and that *mcr* genes and *eptA* share a single common ancestor. Interestingly, the phylogeny constructed here also showed that chromosomally encoded *mcr* genes, which have previously been reported (Ling et al., 2017; Dortet et al., 2018; Stogios et al., 2018; Wang et al., 2021), represent four distinct phylogenetic groups. The chromosomally encoded *mcr* genes of three *Moraxella* spp. were included in subclade II-B-1 (loci numbers AXE82_07515, MC195_01970, and MCR_RS05830; **Figure 3** and **Supplementary Figure S2**). Chromosomally encoded *mcr* genes from *Kosakonia sacchari* (locus number BBB57_02175), *Aeromonas caviae* (locus number WP8S18E04_12720), and *Buttiauxella agrestis* (locus number BADSM9389_18900) grouped into clusters II-B-2e, II-B-2f, and II-B-2f, respectively (**Figure 3** and **Supplementary Figure S2**).

### 3.4 The genomic contexts of *mcr* genes and *eptA* are widely diverse

To identify additional criteria that may aid in *mcr* gene identification, we assessed the genomic context of *eptA, eptB, cptA, mcr*, and the 125 putative novel *mcr*-like genes identified here (**Figure 3**; **Supplementary Figure S2** and **Supplementary Table S11**). Genes in lineage I represented two different genomic contexts: (i) the majority of *cptA* genes in subclade I-B-1 (*n* = 26 out of 33) were located 70-200 nucleotides upstream of an AraC-type transcriptional regulator-encoding gene, while (ii) all 71 *cptA*-related genes in subclade I-B-2 were represented by single gene operons (**Figure 3**; **Supplementary Figure S2** and **Supplementary Table S11**). In comparison, genes in clade II-A (which represented *eptB*; *n* = 66) were always represented by single-gene operons (**Figure 3**; **Supplementary Figure S2** and **Supplementary Table S11**).

The 99 putative novel *mcr*-like genes that clustered with *eptA* into cluster II-B-2g, as well as the 59 chromosomally encoded *eptA* genes, represented two genomic contexts that partitioned into two distinct phylogenetic subgroups (g1 and g2) within cluster II-B-2g (**Figure 3** and **Supplementary Figure S2**). All genes in the “g1” subgroup (*n* = 37) were found to be located adjacent to a putative TetR/AcrR family transcriptional regulator (**Figure 3**; **Supplementary Figure S2** and **Supplementary Table S11**). In contrast, all genes in the “g2” group (*n* = 121) were located in an operon with a PmrAB-like two-component sensor histidine kinase-response regulator system (**Figure 3**, **Supplementary Figure S2**, and **Supplementary Table S11**). Importantly, the known *mcr* genes (which were grouped into subclades II-B-1 and II-B-2) showed genomic contexts that were distinct from the genomic context of the *ipet* genes (detailed in the preceding paragraph); genomic context also differed among the *mcr* families (**Figure 3**; **Supplementary Figure S2** and **Supplementary Table S11**). Among the *mcr* genes in subclade II-B-1, *mcr-1* and *mcr-2* alleles were located in a putative operon upstream of PAP2 family lipid A phosphatase-encoding *pap2* (with some *pap2* in proximity to *mcr-2* showing evidence for truncation), while *mcr-6.1* represented a single gene operon. Interestingly, among the *mcr* genes in cluster II-B-2f, *mcr-7.1* was located 125 nucleotides upstream of an operon that contained *pap2*, as well as *dgkA* (which encodes a diacylglycerol kinase), while *mcr-3* alleles were located upstream of *dgkA*. This is of relevance, as it has been shown that *pap*2 and *dgkA* play a role in *mcr-*dependent colistin resistance through recycling and modification of lipid metabolism byproducts (Choi et al., 2020; Gallardo et al., 2020; Purcell et al., 2022). Among the other *mcr* genes in subclade II-B-2, *mcr-8* alleles were located adjacent to, but divergent from, an operon containing a two-component sensor histidine kinase-response regulator, while alleles belonging to the remaining *mcr* families (*mcr-4*, *mcr-5*, *mcr-9.1*, and *mcr-10.1*) were single-gene operons (**Figure 3**; **Supplementary Figure S2** and **Supplementary Table S11**). The genomic context of the 26 putative novel *mcr*-like genes in clade II-B included the following: (i) the 19 genes in cluster II-B-2d, as well as the three genes in cluster II-B-2f, and the gene ACIN3137_RS04810 in cluster II-B-2e were classified as single gene operons; (ii) the two genes in cluster II-B-2c were located within an operon with a *pap*2 gene; and (iii) the EGY13_RS05385 gene in cluster II-B-2e was located in an operon with a PmrAB-like two-component sensor histidine kinase-response regulator system and divergent from a *pap*2 gene (**Figure 3**; **Supplementary Figure S2** and **Supplementary Table S11**). This suggests that the genomic contexts of the 26 putative novel *mcr*-like genes in clade II-B are similar to those of known *mcr* genes.

### 3.5 Structurally and functionally important disulfide bonds are differentially conserved among different MCR and i-PET families

Sequence analysis of EptA, EptB, CptA, and known MCR proteins showed that five disulfide bonds, which were identified in the EptA structure (Anandan et al., 2017), were differentially conserved among different MCR families (**Supplementary Figure S2** and **Supplementary Table S7**). Previously, structural and mutational analyses of the *E. coli* EptC, a CptA homolog, revealed that the inability of EptC to confer colistin resistance was due to the lack of several disulfide bonds (Zhao et al., 2019). Zhao et al. (Zhao et al., 2019) suggested that the disulfide bonds function synergistically to restrain the flexibility of different loops, especially a loop near the active site, which forms part of a potential substrate entry tunnel. Our sequence analysis showed that CptA homologs (i.e., lineage I) did not have any predicted disulfide bonds. In contrast, EptA and EptB homologs (cluster II-B-2g and clade II-A, respectively) were predicted to contain five and three disulfide bonds, respectively (**Supplementary Figure S2** and **Supplementary Table S7**). Interestingly, three predicted disulfide bonds were conserved in MCR-1, MCR-2, and MCR-6 alleles (which group into subclade II-B-1), while five predicted disulfide bonds were conserved in alleles belonging to the other MCR families (**Supplementary Figure S2** and **Supplementary Table S7**). However, the role that disulfide bond number variability plays in the functional diversity of MCR alleles remains unknown. All 26 putative novel *mcr* genes in clade II-B were predicted to have the same five disulfide bonds as EptA (**Supplementary Figure S2** and **Supplementary Table S7**).

### 3.6 Varying levels of colistin resistance have been reported for different *mcr*-harboring strains

**Figure 4:**
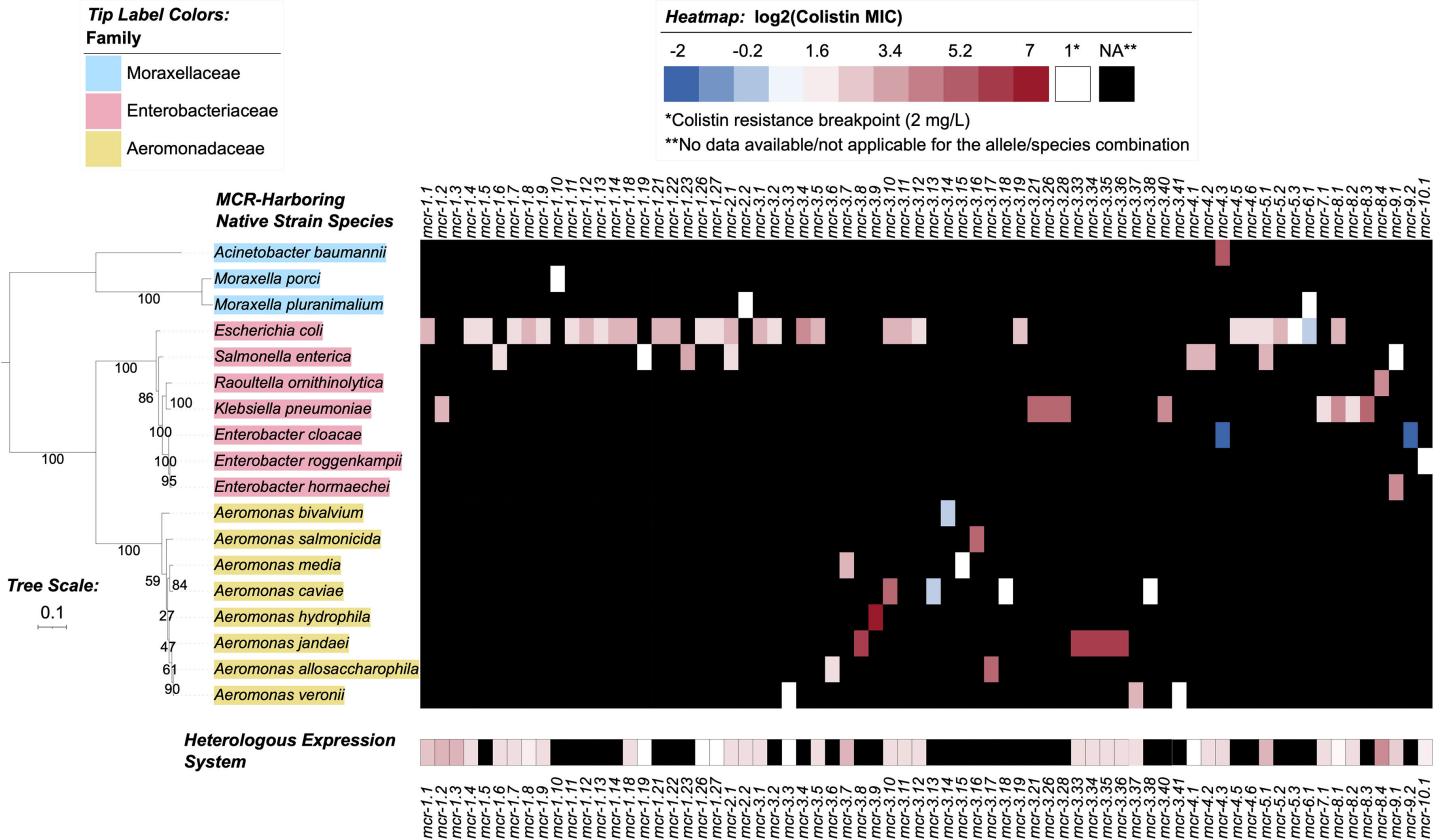
Heatmap showcasing colistin minimum inhibitory concentration (MIC) values reported for MCR alleles in their native strains and in heterologous expression systems. MIC values were obtained from a review of the literature (see the Materials and Methods section for details). Heatmap cell colors correspond to base-2 logarithm-transformed MIC values, where white cells denote a value of 1 (i.e., the log2-transformed colistin resistance breakpoint of 2 mg/L, established by the Clinical and Laboratory Standards Institute [CLSI]); blue shading denotes MIC values below this breakpoint, and red shading denotes MIC values above. MCR alleles with no data reported for a given species (due to the allele not being present in the species, or due to an MIC not being reported) are denoted in the heatmap by black shading. For individual species with multiple MIC values reported for a single MCR allele, the median MIC value is reported (**Supplementary Table S8**). For *mcr-2.1*, *Salmonella enterica* was selected to represent an unspecified “*Salmonella* spp.” reported in the literature for the native strain (**Supplementary Table S8**). The maximum likelihood (ML) phylogeny displayed to the left of the heatmap denotes type strains and/or NCBI representative genomes of reported species for the native strains (**Supplementary Table S8**). Phylogeny tip label colors correspond to taxonomic families assigned via the Genome Taxonomy Database Toolkit (GTDB-Tk). The phylogeny was constructed with IQ-TREE, using an alignment of amino acid sequences produced via GTDB-Tk as input. The phylogeny is rooted along the midpoint, with branch lengths reported in substitutions per site. Branch labels denote branch support percentages obtained using the ultrafast bootstrap approximation. The phylogeny was edited and displayed using iTOL.

Through the literature search conducted here, colistin MIC values were obtained for 70 of 98 *mcr* alleles in their native strains, consistent with reports that MIC data are lacking for multiple *mcr* alleles in the NCBI Gene Catalog (see Nang, et al. for a recent extensive literature survey of colistin MIC values associated with different bacterial isolates) (Nang et al., 2019). Using data extracted from published studies, we found that markedly diverse colistin MIC levels have been reported for native strains harboring different *mcr* families, as well as native strains harboring different *mcr* alleles within the same *mcr* family (**Figures 2**, **4** and **Supplementary Table S8**). For example, different colistin MIC values were reported for native strains harboring *mcr-1.10* (MIC=2 mg/L), *mcr-1.4* (MIC= 4 mg/L), *mcr-1.2* (MIC=8 mg/L), and *mcr-1.23* (MIC=10 mg/L; **Figure 4** and **Supplementary Table S8**). Similarly, native strains harboring different *mcr-3* alleles showed a wide range of reported colistin MIC values (1, 2, 4, 8, 16, 32, and 64 mg/L; **Figures 2**, **4** and **Supplementary Table S8**), despite the relatively high genetic similarity of *mcr-3* alleles (i.e., MCR-3 alleles with reported MIC values share 88.7% to 99.8% AA identity and 97.6% to 100% AA similarity). This showcases that sequence similarity alone cannot explain colistin MIC values in *mcr-3*-harboring native strains, thus highlighting the importance of considering *mcr*-mediated colistin resistance holistically (e.g., considering strain background, genomic context, transcription and expression). Furthermore, low levels of colistin resistance (i.e., MIC < 2 mg/L), which would be classified as colistin susceptible under standard testing conditions, were reported for some *mcr*-harboring bacterial strains (e.g., a strain harboring *mcr-3.14*; **Figure 4** and **Supplementary Table S8**) (Shen et al., 2018; Teo et al., 2018).

Varying colistin MIC values were also reported among different genera expressing the same *mcr* allele (**Figure 4** and **Supplementary Table S8**). For example, *E. coli* and *Salmonella* spp. harboring the *mcr-2.1* allele had colistin MIC values of 8 and 4 mg/L, respectively (Xavier et al., 2016; Garcia-Graells et al., 2018; Poirel et al., 2018). Interestingly, an *E. coli* wildtype strain harboring the *mcr-3.10* allele on a plasmid had a reported colistin MIC value of 8 mg/L, while *Aeromonas caviae* and *Proteus mirabilis* harboring a chromosomal *mcr-3.10* allele had reported colistin MIC values of 32 mg/L (Wang et al., 2018c). Overall, it is apparent that colistin MIC values can vary between *mcr*-harboring native strains. However, any differences between *mcr* families and alleles should be interpreted cautiously, as additional factors may contribute to a given native strain’s colistin MIC (e.g., additional colistin resistance-conferring mutations, the presence or regulation of additional genes affecting colistin sensitivity) (El-Sayed Ahmed et al., 2020).

Notably, multiple *mcr* alleles have also been characterized via heterologous expression in standard laboratory strains (**Figure 4** and **Supplementary Table S8**). MIC values extracted from the literature also indicated extensive variability in colistin MIC values among *E. coli* laboratory strains heterologously expressing different *mcr* alleles (**Supplementary Table S8**). In heterologous expression systems, several factors are likely to affect AMR levels, including the strains’ genetic background, plasmid copy number, level and regulation of gene expression, and cellular toxicity resulting from protein overexpression (Wagner et al., 2008; Schlegel et al., 2012; Tietgen et al., 2018).

### 3.7 Recombination plays a limited role in the evolution of *mcr*

Thirteen potential recombination events were initially identified in an NT_btn_-MSA of *mcr*, putative novel *mcr*-like, and *ipet* genes via multiple detection methods in RDP5 (*n* = 460 total PET-encoding genes; **Supplementary Figure S3** and **Supplementary Table S7**). Specifically, initial evidence for recombination among *mcr-3* alleles was identified (i.e., events 1-3; **Supplementary Figure S3**). However, visual inspection of the recombination events indicated that recombinant fragments in these three events shared similar breakpoints, indicating that these fragments represented a single recombination event (**Supplementary Figure S3**). Moreover, within the *mcr* and *ipet* gene phylogeny, *mcr-3* alleles identified in recombination events 1-4 clustered closely into subclades with relatively short terminal branches (**Figure 3** and **Supplementary Figure S3**). Similar patterns were observed for alleles associated with recombination events 5-8 and 10-13 (**Figure 3** and **Supplementary Figure S3**), indicating that the recombination signals identified in events 1-8 and 10-13 (i.e., all events except for event 9) might have been caused by evolutionary processes other than homologous recombination, such as inter-lineage and inter-site mutation-rate variation (Bertrand et al., 2016; Martin et al., 2021).

Recombination event 9, which included *mcr-7.1*, *mcr-3.34*, and a newly identified putative novel *mcr*-like gene from *Aeromonas caviae* (gene locus ID C1C91_01450 and NCBI Nucleotide accession CP025706.1), was identified by six different recombination detection methods in RDP5 (pairwise homoplasy index [PHI] raw *P* = 0.035; **Supplementary Figure S3**). The PET phylogenies (**Figures 2**, **3**) revealed that the putative recombinant and parents were divergent and had relatively long terminal branches, suggesting that recombination event 9 may have played a role in the evolution of some *mcr-3* and *mcr-7* alleles (**Supplementary Figure S3**). Overall, these results posit a limited role for homologous recombination in the evolution and diversification of *mcr* alleles. However, it is feasible that some recombination events (e.g., between closely related sequences or genes not included in the analysis) were involved in the evolution of *mcr.*

### 3.8 Positive selection likely contributed to the evolution of specific amino acid residues that may induce localized structural variation among MCR alleles

The role of positive selection in the evolution of genes can be assessed by estimating the non-synonymous to synonymous substitution rate (*dN*/*dS*) ratio (w), where an overall w > 1 indicates positive selection (Anisimova and Kosiol, 2009; Delport et al., 2009). Alternatively, w = 1 or w < 1 suggests neutral or negative selection, respectively (Murrell et al., 2012). Overall, an average w value of 0.198 across the 98 known *mcr* alleles was observed (via DnaSP; **Supplementary Table S6**), suggesting that, overall, *mcr* evolved under negative selection. To further test for selection at specific AA sites, we used FUBAR (the Fast, Unconstrained Bayesian AppRoximation method, which infers *dN*/*dS* on a per-site basis) (Murrell et al., 2013), which predicted that 216 of 593 AA sites showed strong evidence for evolving under negative selection (FUBAR posterior probability ≥ 0.99; **Supplementary Figure S4** and **Supplementary Table S12**). We found that AA sites that may have evolved under negative selection were distributed among the three structural components of MCR-1. Specifically, 36% of the AA residues of the membrane-anchored domain, 18% of the AA residues of the bridging region, and 44% of the AA residues of the catalytic domain were predicted to evolve under negative selection (**Supplementary Figure S4**). Using FUBAR, no sites were predicted to evolve under positive selection at posterior probability ≥ 0.99.

**Figure 5:**
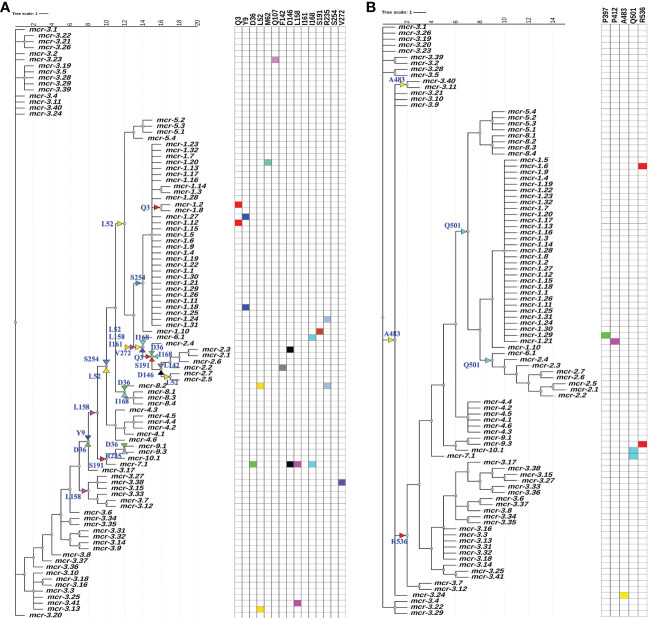
Identification of amino acid (AA) residues under branch-specific positive selection. A phylogeny of 98 known *mcr* alleles produced and partitioned by GARD (https://www.datamonkey.org/gard) shows partition 1 **(A)** and partition 2 **(B)** branch-specific AA sites evolving under positive selection. Sequences were aligned using MUSCLE, and the resulting multiple sequence alignment (MSA) was supplied as input to GARD. The partitioned dataset produced by GARD was supplied as input to MEME (mixed-effect model of evolution-based selection analysis; https://www.datamonkey.org/meme), which was used to identify AA residues under branch-specific positive selection. The tree (default output/rooting produced by GARD/MEME, with branch lengths reported in substitutions per site) was edited using the iTOL web server (https://itol.embl.de/). Color-coded regions and triangles represent AA residues under allele-specific and branch-specific positive selection, respectively.

While an overall *dN*/*dS* ratio < 1 indicates that positive selection did not likely contribute to the overall evolution of *mcr*, positive selection may have still played a role in the evolution of allele-specific and branch-specific AA sites. Indeed, relatively few genes with *dN*/*dS* ratio > 1 are expected to exist (Murrell et al., 2012). Hence, we used a mixed-effect model of evolution (MEME) approach to identify positive selection at the level of individual alleles, a branch, or a set of branches. MEME showed that positive selection may have played a role in the diversification of 19 allele-specific AA sites within 34 branch-specific nodes distributed among two partitions in the phylogeny of 98 known *mcr* alleles (MEME raw *P* < 0.1; **Figure 5** and **Supplementary Table S13**). Among the 19 AA sites, 11 residues were located within the membrane-anchored domain, one in the bridging helix, one in the interdomain flexible loop, and six in the catalytic domain (**Figure 5**). Interestingly, MEME identified multiple positive selection events in the *mcr-2* and *mcr-9* family phylogeny branches (**Figure 5**).

**Figure 6:**
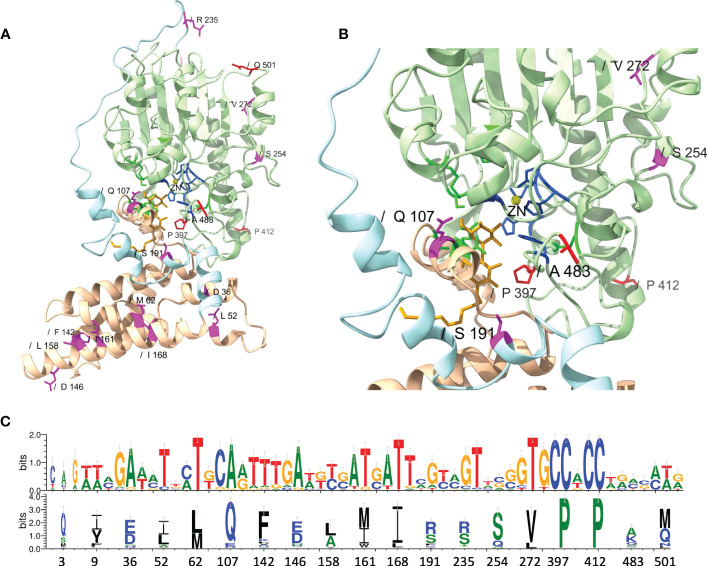
Structural localization and conservation of amino acid (AA) residues under branch-specific positive selection. **(A)** Structural model of the MCR-1 protein, constructed based on *Neisseria meningitidis* phosphoethanolamine transferase EptA (Anandan et al., 2017). The MCR structural model was constructed using the Phyre2 server, and the structure was viewed and edited using UCSF ChimeraX. The structural model shows the transmembrane-anchored domain (yellow) and the soluble periplasmic catalytic domain (light green) connected by a bridging helix and extended loop (light blue). AA residues involved in zinc (yellow circle) and phosphoethanolamine (pEtN) binding are colored dark blue and green, respectively. Branch-specific AA residues evolving under positive selection are shown in magenta (partition 1) and red (partition 2). **(B)** Close-up of the MCR active site showing the localization of branch-specific AA residues under positive selection in relation to the active site and the substrate entry tunnel. The structure shows Gln^107^, which is located at a periplasmic loop connecting the third and fourth transmembrane segments juxtaposing the substrate entry tunnel; Ser^191^, which is located at a bridging region that connects the N-terminal membrane domain and the C-terminal catalytic domain; and Pro^397^, which is located adjacent to the active site. **(C)** Web-logo of nucleotide and AA sequences showing the conservation of 19 AA residues identified by MEME (https://www.datamonkey.org/meme) to have evolved under positive selection among the 98 known MCR alleles.

While MEME did not identify evidence of positive selection for AA residues involved in zinc and pEtN binding, the analysis predicted that several structurally critical residues evolved under positive selection (**Supplementary Table S13**). Mapping of these residues on the structural model of MCR-1 (**Figures 6A, B**) indicated that several of the AA residues predicted to have evolved under positive selection were located in structurally important regions, including: (i) Gln^107^, which is located at a periplasmic loop connecting the third and fourth transmembrane segments juxtaposing the substrate entry tunnel; (ii) Ser^191^ and Arg^235^, which are located at a bridging region that connects the N-terminal membrane domain and the C-terminal catalytic domain; and (iii) Pro^397^and Pro^412^, and Ala^483^, which are located adjacent to the active site and substrate entry tunnel, respectively (**Figures 6A, B**). Subsequently, we evaluated the sequence diversity of the codons associated with these 19 AA residues among the 98 *mcr* alleles (**Figure 6C**). Overall, we found that the coding sequences of the 19 AA residues were highly conserved among alleles of the same *mcr* family. In contrast, these sequences were less conserved among alleles of different families. Specifically, 47 of 57 nucleotide sites were polymorphic among the 98 *mcr* alleles; p values of synonymous and non-synonymous substitutions were 0.472 and 0.348 among all 98 alleles. Comparatively, p values of synonymous and non-synonymous substitutions were 0.003 and 0.017 among *mcr-1* alleles, and 0.028 and 0.029 among *mcr-3* alleles, respectively.

### 3.9 Novel MCR alleles and families may be present among publicly available bacterial genomes

To further assess the novelty of the 125 putative novel *mcr*-like genes identified here, all MCR, putative novel MCR-like, and i-PET proteins were partitioned into clusters based on AA identity (**Supplementary Table S7**). At an 80% AA identity threshold, MCR, putative novel MCR-like, and i-PET AA sequences were partitioned into 64 clusters; of these 64 clusters, the AA sequences associated with the 125 putative novel *mcr*-like genes (**Supplementary Table S3**) were partitioned into 13 clusters, none of which contained known MCR proteins. Considering AA identity among the putative novel MCR-like proteins alone (i.e., ignoring genomic context, as well as AA identity/phylogenetic similarity to i-PET proteins/*ipet* genes), these 13 clusters can be thought of as “PET families”, as 80% AA identity is a conservative MCR family delineation threshold. For comparison, the ten known MCR families were partitioned into seven families using this approach: one family aggregated all MCR-1, MCR-2, and MCR-6 alleles, one aggregated all MCR-9 and MCR-10 alleles, and each of the five remaining known MCR families were partitioned into separate PET families at 80% AA identity. However, when (i) genomic context and (ii) AA identity/phylogenetic similarity to i-PET proteins/*ipet* genes were considered, eight of the 13 PET families (representing 99 putative novel MCR-like proteins) were more similar to EptA than to MCR proteins (i.e., they grouped into cluster II-B-2g; **Figure 3** and **Supplementary Figure S2**). Thus, putative novel MCR-like proteins in cluster II-B-2g are likely to function as EptA or EptA-like proteins rather than *bona fide* MCR proteins.

Notably, five PET families (encompassing 26 putative novel MCR-like proteins) resembled known MCR families, not only by AA identity and phylogenetic relatedness, but also based on genomic context (**Figure 3** and **Supplementary Figure S2**). Indeed, two putative novel *mcr* genes in cluster II-B-2c were located in an operon with a PAP2-encoding gene (**Figure 3** and **Supplementary Figure S2**). Interestingly, the genomic context of a gene in cluster II-B-2e shared characteristics of both *mcr* and *eptA* genomic contexts (locus EGY13_RS05385; **Figure 3** and **Supplementary Figure S2**). While the EGY13_RS05385 gene was located in an operon with a two-component sensor histidine kinase-response regulator system (similar to *eptA* genes), it was located adjacent to a PAP2-encoding gene (similar to *mcr* genes). Thus, a conservative 80% AA identity threshold for clustering not only further supports that we likely identified 26 novel *mcr* genes, but also suggests that these *mcr* genes represent five novel MCR families (designated novel MCR families A to E; **Figure 3**).

As no hard AA identity cutoff has been proposed for defining novel MCR families (Partridge et al., 2018), there may be some ambiguity as to whether the five putative novel MCR-like families identified here truly represent putative novel MCR families or alleles of existing families, despite sharing < 80% AA identity with all known MCR families. For example, the *mcr*-like genes grouped into *mcr* family A (i.e., *Xanthomonas campestris* locus tag BHE83_RS21235 and *Xanthomonas phaseoli* locus tag XASLMG9055_RS15450) were phylogenetically close to *mcr-5*; thus, they might be considered to be alleles of *mcr-5*, rather than a novel *mcr* family (**Figure 3** and **Supplementary Figure S2**). However, in contrast to *mcr-5* alleles, *mcr* family A genes were located in an operon with a PAP2 family lipid A phosphatase-encoding gene, which might suggest that these genes represent a novel *mcr* family, rather than novel *mcr-5* alleles. Similarly, *mcr*-like genes grouped into *mcr* family E (i.e., *Aeromonas hydrophila* and *Aeromonas caviae* locus tags BFW41_RS14915, C1C91_RS01450, and C1C92_RS20765) may be considered to be novel *mcr-7* alleles based on phylogenetic similarity (**Supplementary Figure S2**). However, while the *mcr-7.1* allele was localized adjacent to an operon containing DgkA and PAP2 enzymes, *mcr* family E genes consist of single-gene operons. Comparatively, the *mcr*-like genes classified into *mcr* families B, C, and D (*n* = 6, 13, and 2 genes; **Figure 3**) were phylogenetically distinct from known *mcr* genes but resembled *mcr* genes rather than *eptA* in terms of sequence similarity, phylogenetic relatedness, and genomic context, and thus likely represent novel *mcr* families. Regardless, future experimental and phenotypic analyses are needed to define the potential roles of these genes in colistin resistance and assess their functional diversity.

## 4 Discussion

Overall, our data show that (i) PET proteins can be detected across a wide range of Gram-negative bacterial species and are genetically and functionally diverse; (ii) the evolution and diversification of *mcr* is likely the result of a multifaceted process, which includes positive selection, gene mobilization, and diversification of regulatory pathways and gene contexts; (iii) a holistic approach that considers genomic context, in addition to sequence similarity and genomic localization, may aid in the identification of novel *mcr* families and alleles.

### 4.1 Genetically and functionally diverse PET proteins can be detected across a wide range of Gram-negative bacterial species

Previous studies have shown that PET-encoding genes are distributed across a range of Gram-negative bacteria (Nang et al., 2019; El-Sayed Ahmed et al., 2020; Khedher et al., 2020). Here, we identified >69,000 proteins homologous to colistin resistance-conferring PET in >1,000 bacterial species (based on the mOTUs taxonomy; **Supplementary Table S2**). Using a subset of PET-encoding genes composed of (i) all 98 known MCR-encoding alleles, (ii) 125 genes encoding putative novel MCR-like proteins, and (iii) 237 chromosomal *ipet* genes, we provide further insight into the genetic diversity of colistin resistance-conferring and intrinsic PET-encoding genes alike (**Figure 3**). However, it is important to note the limitations of the criteria used here to identify the 125 putative novel *mcr*-like genes (i.e., co-localization on the same contig as a predicted plasmid replicon and at least one other AMR gene). Previous studies have shown that 10% of bacterial genomes are composed of several essential replicons, including secondary chromosomes and chromids, which use a plasmid-like origin of replication for propagation (Harrison et al., 2010; Dicenzo and Finan, 2017). Hence, the approach used here could incorrectly classify such a contig as plasmid-associated. However, by considering additional factors (e.g., genomic context), we were able to refine our predictions and identify which of the 125 putative novel *mcr*-like genes likely represent *bona fide* novel *mcr* genes. Thus, we believe this limitation has no major impact on our conclusions.

Consistent with previous studies (Carroll et al., 2019; Khedher et al., 2020), we have shown that *mcr* families are genetically diverse. The phylogenetic analyses conducted here also suggest that *eptA* and *mcr* genes share a common ancestor. While previous studies have hypothesized that *mcr* evolved from *eptA* through the mobilization of an *eptA* gene copy flanked by transposable insertion elements (Kieffer et al., 2017; Wang et al., 2018a), with *Moraxella* spp. suggested as a potential source (Kieffer et al., 2017), our data fail to provide clear evidence of a specific event or events that may have led to the emergence of PET genes that confer colistin resistance.

In addition to being genetically diverse, increasing evidence suggests that PET-encoding genes are functionally diverse (Anandan and Vrielink, 2020a). Additionally, it has been shown that different *mcr* alleles may confer different levels of colistin resistance (Nang et al., 2019). While this conclusion is supported by the high level of genetic diversity and the diverse genomic contexts of *mcr* genes (e.g., presence of regulatory genes and genes encoding accessory enzymes that support MCR function, such as PAP2 and DgkA enzymes), assessment of colistin resistance levels in *mcr*-harboring strains should be interpreted with extreme caution. Colistin’s primary mode of action is the displacement of LPS-stabilizing cations, followed by insertion into the membranes and cell disruption; however, it has been suggested that oxidative damage plays a role in colistin-dependent cell killing (Yu et al., 2015; El-Sayed Ahmed et al., 2020). Thus, variations among stress response systems can alter the susceptibility of bacterial cells to colistin treatment (Touati, 2000; Imlay, 2003; O'connor and Mcclean, 2017). Furthermore, additional factors may influence colistin MICs associated with *mcr*-harboring native bacterial strains, including additional colistin resistance-conferring mutations, the level and regulation of *mcr* transcription, the presence or regulation of additional genes affecting colistin sensitivity, and the growth and testing conditions used to assess colistin sensitivity (Martinez-Servat et al., 2018; El-Sayed Ahmed et al., 2020; Loose et al., 2020). Thus, a clear understanding of the extent to which *mcr* genetic diversity contributes to colistin resistance heterogeneity remains a major challenge.

### 4.2 Evolution and diversification of *mcr* is the result of a multifaceted process

Our data suggest that multiple factors might be involved in the evolution and functional diversification of *mcr*, including the diversification of the genomic context and the regulatory control of gene expression. We found that all analyzed chromosomal *eptA*, as well as the putative novel *mcr*-like genes most closely related to *eptA*, were localized adjacent to a TetR-type regulator or a two-component sensor histidine kinase-response regulator system. With the exception of *mcr-8*, no specific regulator was found to be associated with the remaining *mcr* families. In contrast, we found that lipid A phosphatase-encoding *pap2* and diacylglycerol kinase-encoding *dgkA* were frequently located adjacent to many *mcr* alleles. Specifically, *mcr-1* and *mcr-2* alleles were located adjacent to *pap2*, while *mcr-3* and *mcr-8* alleles were located adjacent to *dgkA*. Recently, a detailed mutational analysis indicated that *pap2* and *dgkA* play a significant role in *mcr*-dependent colistin resistance, possibly through recycling and modification of lipid metabolism products byproducts (Choi et al., 2020; Gallardo et al., 2020; Purcell et al., 2022).

Moreover, it was shown that a transposon insertional element has horizontally transferred the *mcr-1*-*pap2* region into different plasmid backbones (Li et al., 2016; Abuoun et al., 2017; Zhang et al., 2019). The role of *pap2* in *mcr* function is further corroborated by the recent identification of a *Sutterella wadsworthensis* gene encoding a single polypeptide with an N-terminal PAP2 domain and C-terminal MCR-like domain (Andrade et al., 2021). Hence, it is evident that the genomic contexts of *eptA* and *mcr* include differential association with the presence of regulatory proteins or accessory enzymes of lipid metabolism. *eptA* genes are exclusively located adjacent to transcription regulators, while *mcr* genes are frequently located adjacent to enzymes for the recycling and modification of lipid metabolism byproducts. These findings may not only help identify novel genes that are likely to represent MCR, but also may provide a framework for further classification of *mcr* genes.

Additionally, our data suggest that positive selection likely contributed to the diversification of specific *mcr* alleles, as we found that positive selection might have played a role in the evolution of several AA residues adjacent to functionally and structurally important protein regions. The AA residues predicted to evolve under positive selection were not part of the active site and the substrate-binding site; however, they are likely to affect enzyme function, such as possible alteration of substrate accessibility, specificity, and enzyme efficacy. For example, we predicted that branch-specific positive selection might be involved in the diversification of Ser^191^ and Arg^235^ in the interdomain-connecting region, which is relevant, as it has been shown that the conformational flexibility of EptA is essential in enzyme-substrate recognition (Anandan et al., 2021). The conformational changes of EptA are governed by a highly conserved domain structure that offers extensive flexibility between the membrane-bound and the periplasmic catalytic domains (Anandan et al., 2017; Anandan and Vrielink, 2020a). Moreover, Anandan et al. (Anandan et al., 2021) proposed that the bridging helix acts as a hinge region that enables extensive conformational changes between the two domains, “opening up” the catalytic domain to allow access to the considerably large lipid A substrate. Hence, diversification of Ser^191^ and Arg^235^ is likely to influence the overall conformational flexibility of the enzyme. For example, at position 191, the relatively small polar Ser (present in *mcr-1* alleles) may offer better conformational flexibility compared to other AA residues identified by MEME at the same locus, such as the non-polar aromatic Phe (*mcr-2* alleles) and the negatively charged Asp (*mcr-8* alleles; **Supplementary Table S13**).

Similarly, the diversification of Gln^107^, Pro^397^, Pro^412,^ and Ala^483^ residues, which are located in periplasmic loops juxtaposed and adjacent to the substrate entry tunnel and the active site (Anandan et al., 2017; Anandan and Vrielink, 2020a) and were also identified as being under positive selection, is likely to affect enzyme activity or specificity by influencing access to the substrate entry tunnel. While our data indicate that diversification of specific AA residues might contribute to the functional diversity of MCR alleles in colistin resistance, detailed site-directed mutational analyses will be required to define the role of positive selection in the functional diversity of MCR. Overall, our data raise the intriguing possibility that *eptA* can give rise to colistin resistance genes through a multifaceted process, which includes positive selection, as well as gene mobilization, diversification of genomic context and regulatory pathways, and possibly (to a more limited extent) recombination. However, the relative contribution of these factors in the evolution of *mcr* remains unknown.

### 4.3 A holistic approach that considers sequence, structure, genomic localization, and genomic context may aid in the discovery of novel *mcr* families and alleles

The identification of novel *mcr* genes is important from both a public health and clinical perspective. For example, in order to monitor and combat the spread of colistin resistance, it is necessary to identify novel *mcr* genes so that they can be included in AMR surveillance efforts (Yin et al., 2017; Carroll et al., 2019). Similarly, a comprehensive database of *mcr* genes can be utilized to screen pathogens isolated from patient clinical samples (e.g., to predict colistin treatment outcome and identify potential treatment failure events).

In addition to providing insight into the genetic and functional diversity of *mcr* and other PET-encoding genes, we identified 125 unique, putative novel *mcr*-like genes, which were located on the same contig as (i) ≥1 plasmid replicon and (ii) ≥1 additional AMR gene. These putative novel MCR-like proteins represented 13 PET families at a conservative 80% AA identity threshold (although there is no strict identity cutoff for defining novel MCR families) (Partridge et al., 2018). Notably, five of these families (representing 26 of the 125 genes) resembled MCR proteins based on AA identity, phylogenetic relatedness, and genomic context, and thus may represent novel MCR families. It may be tempting to speculate on the colistin resistance-conferring potential of these 26 putative novel *mcr*-like genes; however, the discrimination between colistin resistance-conferring *mcr* and intrinsic lipid modification *eptA*, as well as functional assessment of *mcr* alleles, remain a clear challenge, at least partially due to (i) the high genetic diversity among *mcr* families and alleles; (ii) the absence of functional characterization for many *mcr* alleles; and (iii) the extensive diversity of the genetic background of *mcr*-harboring species. While it thus is possible that some of the putative novel *mcr*-like genes identified here may encode i-PET enzymes, we believe that the robust approach used here to identify five putative novel *mcr* families composed of 26 putative novel *mcr* genes minimizes this possibility. However, future functional analyses will be essential to assign biological functions to the 26 putative novel *mcr* genes identified here.

As demonstrated here, comprehensive bioinformatic approaches are necessary to facilitate the initial identification of AMR genes, as inferring AMR phenotypes from bacterial WGS data is often challenging (Tyson et al., 2015; Ransom et al., 2020). In the case of *mcr* and *eptA*, sequence similarity alone is not sufficient to distinguish one from the other, as demonstrated here. However, functional analyses of colistin resistance are also challenging. For example, it has been shown that heterologous overexpression of *Pseudomonas aeruginosa eptA* (Cervoni et al., 2021) or dysregulation of *Salmonella enterica* serotype Typhimurium *eptA* transcription (Vaara et al., 1981; Olaitan et al., 2014) can also lead to colistin resistance. In addition, some *mcr* genes may be expressed under physiologically relevant conditions, but not under standard AMR testing conditions (Kananizadeh et al., 2022). Therefore, functional discrimination between *eptA* and *mcr* may require comprehensive physiological characterization, which may involve cloning in a heterologous expression system and the construction of gene-specific mutants (Gao et al., 2016; Liu et al., 2016; Carroll et al., 2019). These approaches are time-consuming and require a hard-to-achieve standardization process to ensure similar expression levels of active protein and minimal cytotoxicity (resulting from the expression of the membrane-anchored MCR proteins, which is likely to interfere with MIC determination). Identification of additional criteria that can be used to select and prioritize “high risk” *mcr* and *mcr*-like genes for future experimental validation efforts, as reported here, is thus important. Here, we used a high-throughput *in silico* approach to compile a list of >69,000 MCR and MCR-like proteins in publicly available genomes. From this list, we identified 125 unique putative novel *mcr*-like genes, which were refined to 26 *bona fide* putative novel *mcr* genes representing five putative novel *mcr* families, which are candidates for further characterization. Overall, in the future, we imagine that the holistic approach used here, which considers multiple criteria (e.g., sequence similarity, phylogenetic relatedness, genomic context, and structural homology), will facilitate the further identification of novel *mcr* families and alleles from bacterial WGS data and aid researchers in prioritizing *mcr*-like genes for experimental validation.

## Data availability statement

The datasets presented in this study can be found in online repositories. The names of the repository/repositories and accession number(s) can be found in the article/**Supplementary material**.

## Author contributions

AG and LC performed all computational analyses. AG, MW, and LC designed the study and co-wrote the manuscript. All authors contributed to the article and approved the submitted version.
