# Supplementary figures and images for: More than mcr: canonical plasmid- and transposon-encoded mobilized colistin resistance genes represent a subset of phosphoethanolamine transferases
Source: Front Cell Infect Microbiol. 2023 Jun 8;13:1060519. doi: 10.3389/fcimb.2023.1060519 (PMC10285318; doi:10.3389/fcimb.2023.1060519)

Tree scale: 0.1

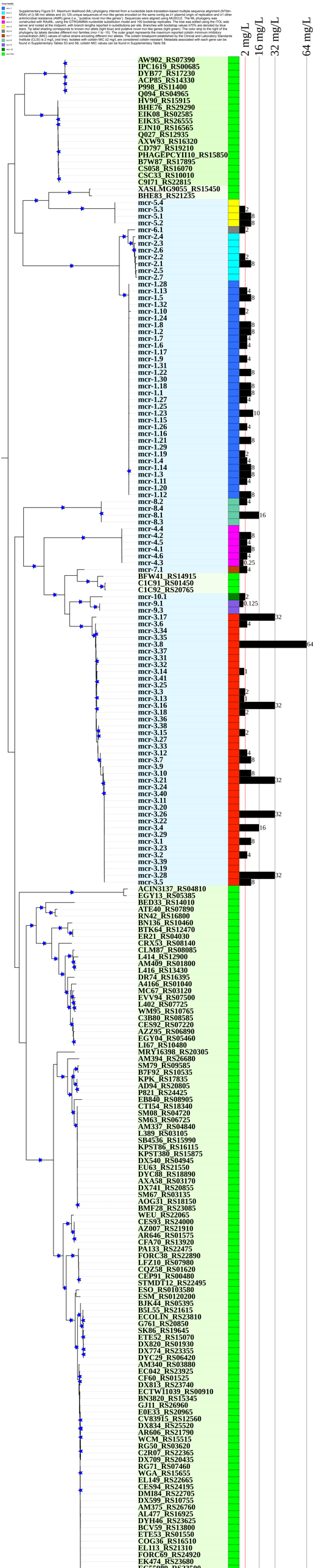

Supplement: Supplementary file 1 [file Image_1.pdf]

Tree scale: 1 

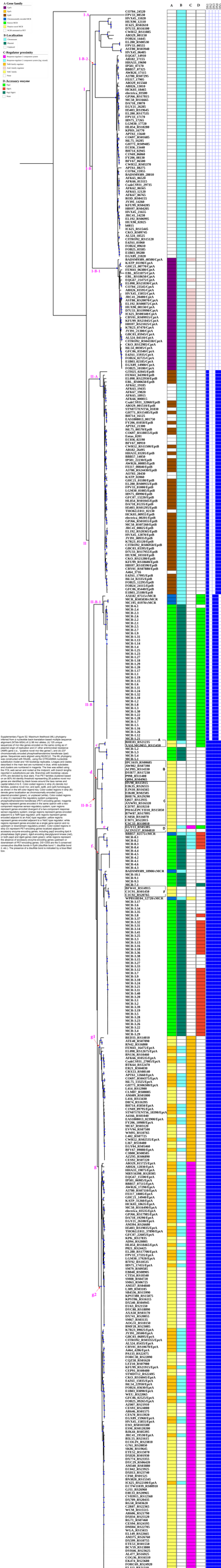

Supplement: Supplementary file 2 [file Image_2.pdf]
