# Supplementary material for: More than mcr: canonical plasmid- and transposon-encoded mobilized colistin resistance genes represent a subset of phosphoethanolamine transferases
Source: Front Cell Infect Microbiol. 2023 Jun 8;13:1060519. doi: 10.3389/fcimb.2023.1060519 (PMC10285318; doi:10.3389/fcimb.2023.1060519)
Supplement: Supplementary file 3 [file Image_3.pdf]

Supplementary Figure S3. Genes with evidence of homologous recombination as detected by RDP5 and PHI analysis.

Event #	Sequence	RDP	GENE-CONV	Boot- scan	Maxchi	Chim-aera	SiSscan	3Seq	PHI p value
1-3[$]	*mcr*-3.33/*mcr*-3.9/*mcr*-3.10[R]<br>*mcr*-3.12/*mcr*-3.14/*mcr*-3.7[Mj]<br>*mcr*-3.3/ *mcr*-3.13/ *mcr*-3.16 [Mn]	1.03E-26	3.09E-23	2.44E-27	2.09E-13	9.61E-14	1.44E-21	4.62E-41	<0.00001
4[$]	*mcr*-3.1[R]<br>*mcr*-3.37[Mj]<br>*mcr*-3.17[Mn]	NS	3.7E-18	4.9E-22	1.7E-11	1.1E-11	6.6E-16	6.4E-25	<0.00001
5	PET_IC625_RS02610[R]<br>*yhbX*_D7U33_RS16100[Mj]<br>PET_HUX90_12110[Mn]	NS	5.0E-09	6.5E-12	6.5E-11	1.4E-10	7.9E-16	3.3E-21	0.29
6[$]	*mcr*-like_DX820_RS01930[R]<br>*mcr*-like_ETE52_RS15070[Mj]<br>*eptA*_JRC41_19530[Mn]	NS	NS	NS	2.8E-10	2.5E-04	1.7E-10	1.6E-13	<0.00001
7[$]	*mcr*-like_SK86_RS19645[R]<br>*mcr*-like_AM340_RS03880[Mj]<br>*eptA*_JRC41_19530[Mn]	NS	NS	NS	1.9E-03	1.9E-06	8.0E-10	1.2E-12	<0.00001
8[$]	*mcr*-like_P821_RS24425[R]<br>*eptA*_I8N75_17455[Mj]<br>*mcr*-like_EU63_RS21550[Mn]	2.4E-06	8.5E-08	3.5E-07	4.4E-06	3.9E-08	3.7E-07	1.6E-11	<0.00001
9	*mcr*-7[R]<br>*mcr*-3.34[Mj]<br>*mcr*-like_C1C91_RS01450[Mn]	9.25E-07	NS	9.09E-10	4.07E-12	7.16E-15	1.87E-30	2.55E-15	**0.0348**
10[$]	*eptB*_FY206_01050[R]<br>*eptB*_electrica_00201[Mj]<br>PET_BFV67_00910[Mn]	NS	3.9E-09	1.8E-05	4.8E-05	9.9E-04	1.0E-08	NS	3.9E-03
11	*cptA*_HVX45_15055[R]<br>*cptA*_EL192_RS00875[Mj]<br>*cptA*_ D7U33_RS19990[Mn]	4.7E-02	NS	3.8E-02	6.2E-04	2.3E-05	NS	4.9E-06	0.11
12[$]	*mcr*-like_EB840_RS08905[R]<br>*mcr*-like_EU63_RS21550[Mj]<br>*eptA*_I8N75_17455[Mn]	NS	3.2E-03	2.2E-04	5.8E-05	4.3E-05	4.4E-07	NS	<0.00001
13	*cptA*_JRC41_20400[R]<br>*cptA*_GBC03_05945[Mj]<br>*cptA*_ E4Z61_15935[Mn]	NS	NS	2.7E-02	1.4E-03	4.4E-05	2.4E-04	9.8E-03	0.20

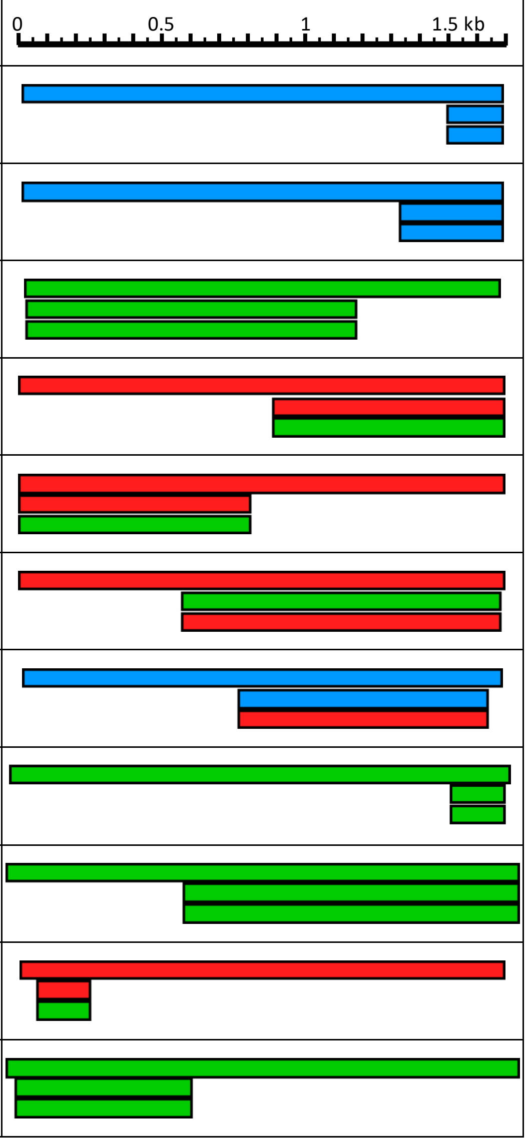

NS: No significant P-value was recorded for this recombination event using this method.
($) RDP analysis of this event indicates that recombination might not have caused this signal, while P-value indicates some evidence of recombination.
R: recombinant; Mj: major parent; Mn: minor parent
